# Spatiotemporal trends of disease burden of edentulism from 1990 to 2019: A global, regional, and national analysis

**DOI:** 10.3389/fpubh.2022.940355

**Published:** 2022-11-28

**Authors:** Xiao Li, Jinyu Man, Hui Chen, Xiaorong Yang

**Affiliations:** ^1^Department of Special Treatment, Jinan Stomatological Hospital, Jinan, China; ^2^Department of Epidemiology and Health Statistics, School of Public Health, Cheeloo College of Medicine, Shandong University, Jinan, China; ^3^Clinical Epidemiology Unit, Qilu Hospital of Shandong University, Jinan, China; ^4^Clinical Research Center of Shandong University, Jinan, China

**Keywords:** edentulism, temporal trend, global disease burden, DALYs, prevalence

## Abstract

**Background:**

Understanding the spatiotemporal trends in disease burden of edentulism is critical to reducing disease burden of edentulism and deploying medical resources. We assessed the changing patterns of disease burden of edentulism at global, regional, and national levels from 1990 to 2019.

**Methods:**

Data on incident cases, prevalent cases, disability-adjusted life years (DALYs), and age-standardized rates (ASRs) of edentulism were extracted from the Global Burden of Disease 2019 Study. We calculated the estimated annual percentage change (EAPC) to quantify spatiotemporal trends in the ASRs of edentulism.

**Results:**

In 2019, the number of prevalent cases and DALYs of edentulism were 35.2 and 9.6 million, and the ASPR and ASDR were 43.12/1,000 and 1.18/1,000, with EAPCs of −0.18 [95% confidence interval (CI): −0.28, −0.09] and −0.16[95% CI: −0.26, −0.07] from 1990 to 2019, respectively. Females and the elderly had a higher burden of edentulism. Although the ASPR, ASDR, and ASIR in the high SDI, high–middle SDI, and middle SDI regions showed a decreasing or stable trend, the absolute disease burdens of edentulism in these regions were still high. Although the absolute disease burdens of low SDI and low–middle SDI were low, their ASPR and ASDR showed an upward trend. In countries with high initial disease burden or high SDI, ASPR, ASDR, and ASIR showed stable or declining trends.

**Conclusion:**

The absolute disease burden due to edentulism was increasing in many countries and regions. Countries should reduce the disease burden caused by edentulism by adopting measures including the prevention and treatment of dental caries and periodontal disease.

## Introduction

Edentulism, defined as the complete loss of all teeth, is a global phenomenon. Tooth loss reflects the complex outcome of the treatment of individual dental disease and its use of dental services in life, and is the ultimate marker of the burden of oral health disease commonly found in older and poorer populations (1, 2). A large amount of dental falling will affect the diet, causing weight loss and in nutrition. In addition, the tooth that falls off may also damage the appearance and hinder communication, thereby restricting social contact, and reducing self-esteem (3). According to the World Health Organization criteria, edentulous patients are considered physically impaired, disabled, and handicapped (4). In addition, in recent years, there have been some studies that edentulism may also be associated with cardiovascular disease, cognitive disorders, dementia, depression, and COPD (5–9). Further, studies have also shown that missing teeth appear to be associated with cancer, and the relationship remains significant after adjusting for socioeconomic status and related factors (10–13). Therefore, we need to fully understand the temporal trend and influencing factors of edentulism, in order to develop targeted public policies to reduce people's disease burden.

In recent years, several studies have assessed the disease burden of edentulism using national data from different countries. Schwendick et al. analyzed the prevalence trends of edentulism in adults and the elderly from 1997 to 2014 based on the data from the German Oral Health Studies and evaluated the predictors of edentulism (14, 15). Wu and Weintraub et al. analyzed the temporal trends and related factors of edentulism of Asian Americans, Aboriginals, and the American elderly (16–18). Kailembo et al. used data from World Health Organization Study on global AGEing and adult health Wave 1 (2007–2010) to analyze the burden and behavioral risk factors of edentulism in four low- and middle-income countries (China, Ghana, India, and South Africa) and found differences in prevalence and risk factors for edentulism between countries (2). Further, some researchers explored and comprehensively reported the global prevalence of oral conditions in 1990–2010 and 1990–2017 in 2013 and 2020, respectively, providing a basis for the prevention and control of oral conditions (19, 20). Marcenes et al. explored the global prevalence and incidence of oral conditions from 1990 to 2010 by gender and age group, and found that the prevalence and incidence of severe tooth loss improved in most places from 1990 to 2010, however, similar improvements have not been achieved in South Asia, Eastern Europe, southern Latin America, Oceania, and central sub-Saharan Africa (1). However, nearly a decade has passed, and the disease burden and temporal trends of edentulism may have changed, and the global estimation which is specific to the disease burden of edentulism has not been reported. It is necessary for us to use new data to update the disease burden and temporal trend of edentulism and analyze the influencing factors of the disease trend so that effective preventive measures can be prioritized to reduce the disease burden of the population under the premise of increasing population aging.

Global Burden of Disease (GBD) 2019 systematically assessed and updated disease burden and influencing factors in 204 countries and territories, providing a unique opportunity to study the influencing factors of the disease burden of edentulism. In this study, we aim to estimate the temporal trends and the influential factors of the disease burden of edentulism by genders, and age groups at global, regional, and national levels. Our findings will provide a basis for policy formulation and medical resource allocation to reduce the global burden of edentulism.

## Materials and methods

Details of GBD studies have been reported in previous studies (21–23), and the method we present here is specific to the disease burden of edentulism estimation. The Guidelines for Accurate and Transparent Health Estimates Reporting were followed at every step of the analysis of the GBD database (24).

### Study data

In our study, annual incident cases, age-standardized incidence rate (ASIR), annual prevalent cases, age-standardized prevalence rate (ASPR), disability-adjusted life years (DALYs), and age-standardized DALY rate (ASDR) of edentulism by 5-year age groups, genders, regions, and risk factors from 1990 to 2019 were collected from the GBD 2019 database *via* the Global Health Data Exchange (GHDx) query tool (http://ghdx.healthdata.org/gbd-results-tool). Edentulism was defined as the absence of any permanent teeth in the mouth; toothlessness in infancy was not included. A total of 204 countries and regions were included in the GBD database and were divided into 5 regions according to the socio-demographic index (SDI), which was calculated by combining educational attainment, total fertility rate, and the lag-distributed income per capita. Further, 204 countries and territories were divided into 21 GBD regions based on geographical proximity and epidemiological similarity, and further simplified into seven Super GBD regions.

Many previous studies have detailed methods for disease burden estimation. In short, based on published literature and cross-sectional studies, DisMod MR 2.1 was used to estimate the disease burden of edentulism, and GBD comparative risk assessment was used to quantify the exposure to risk factors and its attributable burden (21, 22). The GBD study estimated age-standardized rates (ASRs) based on the GBD-standardized population. Relevant data were reported as numbers and 95% uncertainty interval (UI) was determined by 2.5 and 97.5% of the ordered 1,000 estimates.

### Statistical analyses

ASIR, ASPR, ASDR, and estimated annual percentage change (EAPC) were applied to quantify the trends of the disease burden of edentulism by age, gender, regions, and risk factors from 1990 to 2019. Standardization is important for our study because it avoids differences in the age composition of different groups even within the same population at different times. EAPC was a widely used measure that summarizes ASR trends over a specified time interval, and we calculated it to describe the temporal trends of ASRs of edentulism burden. ASR was put in the regression line model “ln (ASR) = α + β^*^calendar year + ε”, and the calculation formula of EAPC as 100 × [exp (β) – 1]. We generated the 95% confidence interval (CI) for EAPC from this model. If both the EAPC estimate and the lower bound of its 95% CI is > 0, the ASR is considered to be in an upward trend. Conversely, if the EAPC estimate and the upper limit of its 95% CI were <0, the ASR was considered to be trending downward. Otherwise, ASR is considered stable (25–29). We used the Spearman rank test to explore the influencing factors of EAPC. We assessed the relationship between EAPC and ASR in 1990 and SDI in 2019 separately at the country level. ASR in 1990 reflects the initial burden of disease, while SDI in 2019 can be used as a proxy for the socioeconomic level and availability of healthcare services in different countries (25, 30, 31). R program was used for all statistical analyses (version 4.0.3; https://www.R-project.org/) and a two-sided *P*-value < 0.05 was considered statistically significant.

## Results

### The global burden due to edentulism

**Figure 1:**
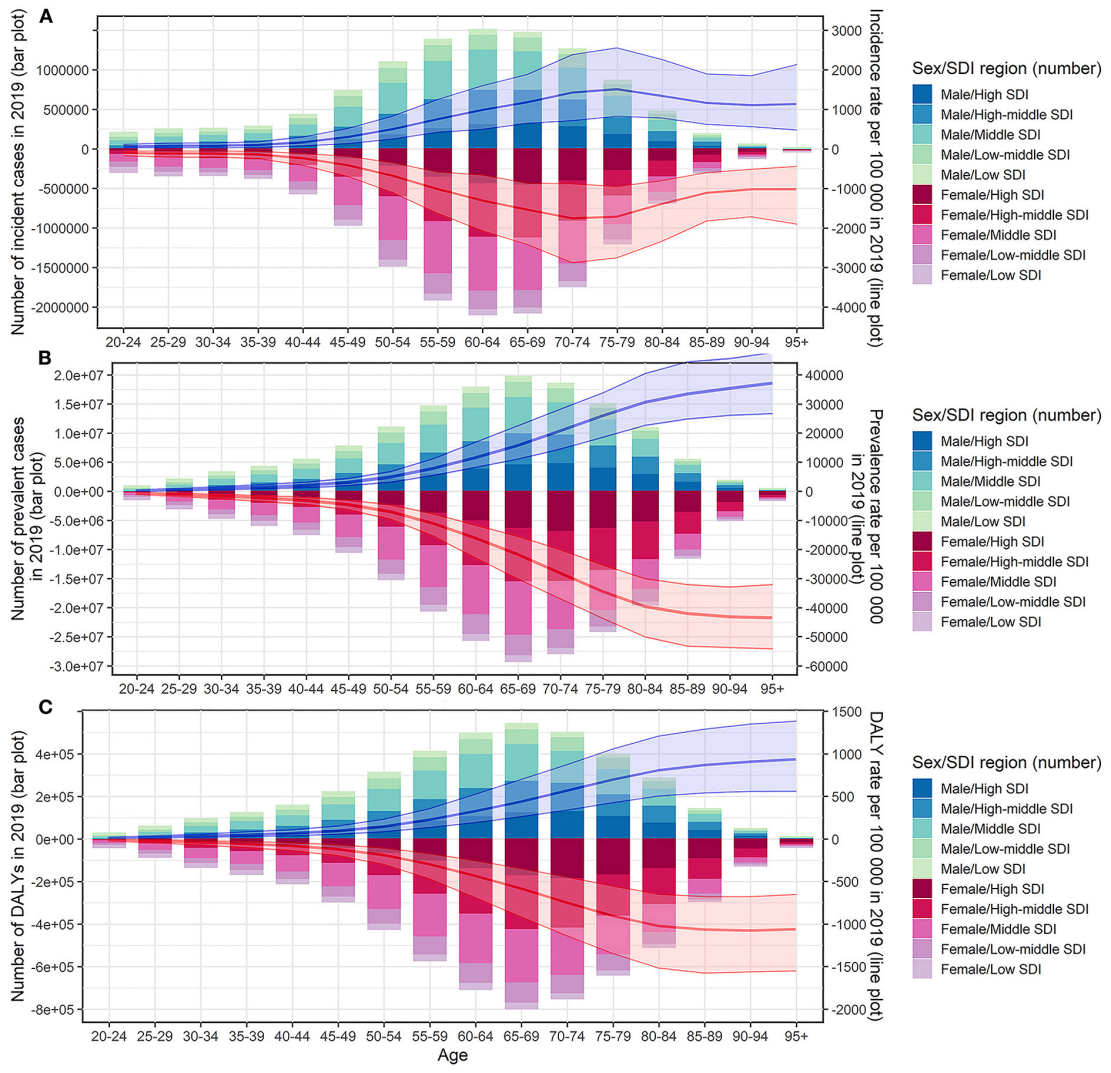
Incident cases, prevalent cases, DALYs and the corresponding rates of edentulism by sex, age group, and SDI regions in 2019. **(A)** Incident cases and incidence rate; **(B)** prevalent cases and prevalence rate; **(C)** DALYs and DALY rate. DALYs, disability-adjusted life years; SDI, socio-demographic index.

Globally, the number of edentulism patients increased from 19.44 million in 1990 to 35.20 million in 2019, and ASPR showed a slight downward trend from 49.36 to 43.12 per 1,000 during the period, with an EAPC of −0.18 (95% CI: −0.28, −0.09). The incident cases of edentulism were 25.00 million in 2019, with a 78.6% increase from 14.03 million in 1990, and ASIR showed a downward trend, with an EAPC of −0.26 (95% CI: −0.36, −0.16). The DALYs of edentulism worldwide increased from 5.32 to 9.62 million over the past 30 years, while the ASDR decreased during the 30 years, with EAPC of −0.16 (95% CI: −0.26, −0.07). Compared with males, the number of incident cases, prevalence cases, DALYs, ASIR, ASPR, and ASDRs of females were larger, and the disease burden of females was heavier, while the EAPCs of males and females were similar (Table 1). Most edentulism patients were between 55 and 79 years old, and the disease burden in those age groups was also heavier (Figure 1).

**Table 1:** Prevalence, DALYs, incidence and age-standardized rate per 1,000 people for edentulism in 1990 and 2019, and its estimated annual percentage change from 1990 to 2019.

	**Prevalence**					**DALYs**					**Incidence**				
	**1990**		**2019**		**EAPC (95%CI)**	**1990**		**2019**		**EAPC (95%CI)**	**1990**		**2019**		**EAPC (95%CI)**
	**ASPR/1,000 (95%UI)**	**Prevalence** *10 **^**∧**^5 (95%UI)**	**ASPR/1,000 (95%UI)**	Prevalence* **10^**∧**^5 (95%UI)**	**From 1990 to 2019**	**ASDR/1,000 (95%UI)**	DALYs* **10^**∧**^5 (95%UI)**	**ASDR/1,000 (95%UI)**	DALYs* **10^**∧**^5 (95%UI)**	**From 1990 to 2019**	**ASIR/1,000 (95%UI)**	Incident cases* **10^**∧**^5 (95%UI)**	**ASIR/1,000 (95%UI)**	Incident cases* **10^**∧**^5 (95%UI)**	**From 1990 to 2019**
**Global**	49.36 (39.50, 62.63)	194.35 (154.59, 248.17)	43.12 (34.47, 54.68)	352.02 (280.34, 449.25)	−0.18 (−0.28, −0.09)	1.34 (0.86, 1.97)	53.23 (34.19, 78.39)	1.18 (0.75, 1.72)	96.22 (61.53, 141.75)	−0.16 (−0.26, −0.07)	3.35 (2.67, 4.08)	140.31 (111.44, 173.35)	3.01 (2.40, 3.69)	250.01 (198.11, 307.39)	−0.26 (−0.36, −0.16)
Male	41.43 (33.10, 52.75)	74.66 (59.03, 96.41)	36.61 (29.16, 46.52)	139.53 (110.45, 179.71)	−0.17 (−0.27, −0.07)	1.13 (0.72, 1.68)	20.59 (13.08, 30.55)	1.00 (0.64, 1.49)	38.40 (24.44, 56.69)	−0.15 (−0.25, −0.05)	2.94 (2.34, 3.64)	57.94 (45.52, 73.08)	2.66 (2.11, 3.30)	105.16 (82.24, 131.77)	−0.26 (−0.37, −0.15)
Female	56.07 (44.93, 71.09)	119.69 (95.71, 151.40)	48.91 (39.13, 61.96)	212.49 (169.87, 269.68)	−0.16 (−0.28, −0.05)	1.52 (0.98, 2.23)	32.64 (21.08, 47.87)	1.33 (0.85, 1.95)	57.82 (37.03, 84.50)	−0.15 (−0.27, −0.03)	3.74 (2.99, 4.52)	82.37 (65.94, 100.17)	3.35 (2.68, 4.07)	144.85 (115.57, 176.42)	−0.26 (−0.38, −0.15)
**SDI region**															
High SDI	54.77 (43.40, 69.68)	56.46 (44.65, 71.72)	46.05 (36.53, 58.91)	81.05 (64.11, 103.01)	−0.79 (−1.19, −0.39)	1.49 (0.96, 2.21)	15.38 (9.84, 22.75)	1.26 (0.80, 1.87)	21.97 (14.01, 32.50)	−0.79 (−1.18, −0.39)	3.68 (2.91, 4.51)	36.73 (29.01, 44.70)	3.12 (2.46, 3.86)	50.6 (39.43, 62.52)	−0.80 (−1.15, −0.45)
High–middle SDI	55.94 (45.28, 70.51)	59.16 (47.42, 75.17)	47.78 (38.03, 60.79)	96.26 (76.16, 123.23)	−0.49 (−0.61, −0.38)	1.53 (0.98, 2.23)	16.20 (10.40, 23.77)	1.31 (0.84, 1.92)	26.32 (16.88, 38.99)	−0.48 (−0.59, −0.36)	3.79 (3.05, 4.57)	42.19 (33.57, 51.47)	3.35 (2.67, 4.10)	67.46 (52.87, 82.90)	−0.44 (−0.58, −0.30)
Middle SDI	47.34 (37.96, 59.81)	48.37 (38.21, 61.59)	44.51 (35.66, 56.16)	109.51 (87.12, 140.37)	0.13 (−0.04, 0.31)	1.29 (0.83, 1.89)	13.34 (8.56, 19.70)	1.22 (0.78, 1.78)	30.08 (19.18, 44.31)	0.15 (−0.02, 0.32)	3.36 (2.69, 4.07)	37.89 (30.23, 47.08)	3.20 (2.57, 3.90)	82.86 (65.63, 102.6)	−0.02 (−0.21, 0.17)
Low–middle SDI	38.26 (30.30, 48.91)	22.47 (17.38, 29.33)	35.35 (27.96, 44.53)	47.82 (37.44, 61.21)	0.70 (0.31, 1.08)	1.03 (0.66, 1.52)	6.15 (3.92, 9.21)	0.96 (0.61, 1.40)	13.05 (8.34, 19.37)	0.73 (0.35, 1.11)	2.74 (2.19, 3.39)	17.72 (13.99, 22.18)	2.56 (2.04, 3.15)	36.55 (29.06, 45.04)	0.22 (−0.15, 0.59)
Low SDI	31.41 (25.02, 39.63)	7.79 (6.10, 10.05)	30.52 (24.19, 38.29)	17.17 (13.51, 22.04)	0.68 (0.37, 0.99)	0.85 (0.54, 1.25)	2.14 (1.37, 3.18)	0.83 (0.53, 1.22)	4.74 (3.04, 6.99)	0.71 (0.40, 1.02)	2.13 (1.70, 2.64)	5.69 (4.51, 7.21)	2.06 (1.65, 2.56)	12.38 (9.83, 15.41)	0.20 (−0.09, 0.50)
**GBD region**															
High-income Asia Pacific	43.29 (34.06, 54.02)	8.54 (6.72, 10.75)	34.44 (27.12, 43.06)	14.81 (11.56, 18.34)	−0.70 (−1.40, 0.00)	1.19 (0.77, 1.75)	2.35 (1.50, 3.46)	0.95 (0.60, 1.40)	4.04 (2.57, 5.94)	−0.68 (−1.38, 0.02)	3.24 (2.58, 3.91)	6.59 (5.19, 8.03)	2.58 (2.04, 3.17)	10.10 (7.88, 12.46)	−0.72 (−1.33, −0.11)
High-income North America	57.74 (43.74, 76.41)	19.70 (14.91, 25.92)	47.60 (37.35, 62.43)	27.17 (21.11, 35.25)	−1.02 (−1.40, −0.64)	1.56 (0.96, 2.35)	5.31 (3.28, 8.01)	1.29 (0.81, 1.92)	7.29 (4.62, 10.76)	−1.02 (−1.40, −0.65)	3.75 (2.88, 4.76)	12.16 (9.37, 15.18)	2.94 (2.29, 3.71)	15.56 (11.92, 19.79)	−1.20 (−1.55, −0.84)
Western Europe	56.45 (45.63, 70.77)	31.94 (25.60, 39.89)	49.22 (38.59, 63.14)	41.54 (32.48, 52.89)	−0.82 (−1.38, −0.26)	1.55 (1.00, 2.27)	8.72 (5.63, 12.82)	1.35 (0.87, 2.02)	11.29 (7.26, 16.84)	−0.82 (−1.38, −0.25)	3.86 (3.14, 4.69)	20.60 (16.60, 24.81)	3.42 (2.65, 4.23)	25.56 (19.86, 31.38)	−0.76 (−1.28, −0.25)
Australasia	102.80 (97.93, 108.10)	2.37 (2.25, 2.49)	65.26 (51.70, 83.45)	3.03 (2.37, 3.86)	−0.72 (−1.30, −0.14)	2.83 (1.93, 3.92)	0.65 (0.44, 0.90)	1.79 (1.14, 2.64)	0.82 (0.52, 1.21)	−0.72 (−1.31, −0.13)	5.70 (5.34, 6.04)	1.28 (1.20, 1.36)	4.22 (3.33, 5.18)	1.80 (1.40, 2.20)	−0.71 (−1.08, −0.34)
Southern Latin America	51.88 (40.57, 66.09)	2.38 (1.86, 3.05)	44.15 (34.30, 56.94)	3.67 (2.85, 4.72)	−0.53 (−0.56, −0.51)	1.42 (0.89, 2.13)	0.66 (0.41, 0.98)	1.21 (0.76, 1.81)	1.00 (0.63, 1.50)	−0.54 (−0.57, −0.51)	3.69 (2.89, 4.56)	1.73 (1.35, 2.15)	3.19 (2.48, 3.98)	2.57 (2.00, 3.19)	−0.48 (−0.50, −0.45)
Andean Latin America	103.75 (85.32, 126.76)	2.22 (1.81, 2.73)	95.15 (77.10, 118.31)	5.40 (4.37, 6.74)	−0.29 (−0.38, −0.19)	2.87 (1.85, 4.15)	0.62 (0.40, 0.90)	2.63 (1.68, 3.82)	1.50 (0.95, 2.19)	−0.29 (−0.39, −0.19)	6.21 (5.20, 7.15)	1.47 (1.24, 1.73)	5.80 (4.78, 6.83)	3.42 (2.80, 4.06)	−0.24 (−0.32, −0.15)
Tropical Latin America	94.60 (76.91, 115.53)	8.73 (7.05, 10.80)	91.29 (73.90, 112.52)	22.24 (17.90, 27.53)	−0.09 (−0.47, 0.28)	2.57 (1.65, 3.72)	2.39 (1.53, 3.51)	2.50 (1.60, 3.64)	6.11 (3.89, 8.93)	−0.08 (−0.46, 0.30)	6.03 (5.06, 6.94)	6.27 (5.22, 7.33)	5.84 (4.84, 6.77)	14.76 (12.17, 17.29)	−0.06 (−0.25, 0.14)
Central Latin America	61.89 (49.26, 79.20)	5.44 (4.33, 6.98)	58.12 (46.13, 74.36)	13.90 (11.03, 17.83)	−0.44 (−0.59, −0.30)	1.69 (1.07, 2.47)	1.50 (0.95, 2.21)	1.59 (1.01, 2.34)	3.82 (2.42, 5.60)	−0.43 (−0.57, −0.29)	3.97 (3.16, 4.87)	3.87 (3.09, 4.78)	3.74 (2.97, 4.62)	9.25 (7.34, 11.57)	−0.42 (−0.55, −0.28)
Caribbean	55.93 (43.74, 71.94)	1.46 (1.14, 1.88)	51.80 (42.88, 63.18)	2.68 (2.22, 3.27)	−0.35 (−0.40, −0.30)	1.54 (0.98, 2.29)	0.40 (0.26, 0.60)	1.42 (0.92, 2.04)	0.73 (0.48, 1.06)	−0.36 (−0.41, −0.32)	3.95 (3.10, 4.89)	1.06 (0.83, 1.31)	3.67 (3.06, 4.37)	1.91 (1.59, 2.28)	−0.33 (−0.37, −0.28)
Eastern Europe	68.48 (55.08, 86.49)	18.65 (15.03, 23.76)	67.38 (54.13, 85.08)	22.42 (17.93, 28.42)	−0.09 (−0.16, −0.02)	1.87 (1.20, 2.73)	5.09 (3.26, 7.47)	1.85 (1.18, 2.71)	6.12 (3.91, 8.98)	−0.07 (−0.14, 0.00)	4.37 (3.52, 5.28)	12.22 (9.57, 14.93)	4.33 (3.47, 5.23)	13.98 (10.86, 17.04)	−0.07 (−0.12, −0.02)
Central Europe	70.40 (55.98, 89.58)	10.24 (8.06, 13.11)	57.38 (45.49, 73.81)	11.71 (9.15, 15.00)	−0.82 (−0.95, −0.69)	1.92 (1.22, 2.82)	2.80 (1.78, 4.15)	1.57 (1.00, 2.33)	3.19 (2.03, 4.73)	−0.81 (−0.94, −0.67)	4.63 (3.69, 5.60)	6.83 (5.34, 8.36)	3.86 (3.06, 4.76)	7.34 (5.70, 9.01)	−0.73 (−0.84, −0.63)
Central Asia	85.74 (69.45, 107.25)	4.07 (3.27, 5.14)	81.01 (65.41, 101.27)	6.11 (4.86, 7.79)	−0.31 (−0.40, −0.23)	2.37 (1.52, 3.46)	1.13 (0.72, 1.67)	2.24 (1.43, 3.27)	1.70 (1.09, 2.51)	−0.32 (−0.41, −0.24)	5.29 (4.31, 6.30)	2.74 (2.21, 3.34)	5.07 (4.09, 6.08)	4.35 (3.43, 5.45)	−0.22 (−0.27, −0.17)
North Africa and Middle East	72.59 (58.16, 91.47)	13.21 (10.51, 16.85)	66.64 (52.85, 84.68)	30.81 (24.53, 39.35)	−0.38 (−0.46, −0.30)	2.00 (1.28, 2.93)	3.67 (2.33, 5.42)	1.83 (1.17, 2.69)	8.54 (5.44, 12.59)	−0.39 (−0.48, −0.31)	4.49 (3.60, 5.44)	9.26 (7.37, 11.42)	4.18 (3.34, 5.12)	21.29 (16.87, 26.43)	−0.31 (−0.38, −0.25)
South Asia	31.59 (23.96, 40.68)	17.29 (12.78, 23.16)	28.83 (22.13, 36.61)	39.49 (29.75, 51.15)	1.71 (0.92, 2.50)	0.84 (0.53, 1.26)	4.68 (2.94, 7.16)	0.77 (0.49, 1.15)	10.65 (6.69, 16.13)	1.79 (0.99, 2.60)	2.48 (1.93, 3.11)	14.58 (11.14, 18.75)	2.24 (1.75, 2.81)	31.97 (24.85, 40.41)	0.49 (−0.14, 1.12)
Southeast Asia	47.00 (37.29, 60.12)	12.04 (9.46, 15.57)	41.84 (33.07, 53.69)	25.20 (19.74, 32.74)	−0.41 (−0.45, −0.37)	1.28 (0.82, 1.89)	3.31 (2.12, 4.89)	1.14 (0.73, 1.69)	6.93 (4.43, 10.31)	−0.40 (−0.44, −0.36)	3.35 (2.63, 4.12)	9.49 (7.48, 11.98)	3.01 (2.36, 3.72)	19.47 (15.08, 24.74)	−0.39 (−0.44, −0.35)
East Asia	34.89 (27.13, 44.23)	28.29 (21.85, 36.79)	33.10 (25.76, 42.03)	66.19 (51.11, 85.50)	0.22 (−0.42, 0.87)	0.95 (0.61, 1.41)	7.78 (4.96, 11.81)	0.90 (0.57, 1.35)	18.10 (11.63, 27.29)	0.25 (−0.40, 0.90)	2.82 (2.22, 3.50)	25.38 (19.50, 31.91)	2.71 (2.14, 3.37)	57.02 (43.76, 72.06)	0.02 (−0.50, 0.54)
Oceania	59.02 (46.86, 74.62)	0.17 (0.13, 0.22)	56.48 (44.63, 71.76)	0.38 (0.30, 0.49)	−0.14 (−0.16, −0.13)	1.60 (1.02, 2.36)	0.05 (0.03, 0.07)	1.52 (0.98, 2.26)	0.10 (0.07, 0.15)	−0.15 (−0.16, −0.13)	4.29 (3.40, 5.22)	0.14 (0.11, 0.18)	4.15 (3.26, 5.07)	0.33 (0.25, 0.41)	−0.11 (−0.12, −0.09)
Western Sub-Saharan Africa	24.36 (19.21, 30.93)	2.32 (1.82, 3.03)	23.07 (18.13, 29.33)	4.98 (3.86, 6.50)	−0.32 (−0.56, −0.07)	0.67 (0.43, 1.00)	0.64 (0.41, 0.96)	0.63 (0.40, 0.95)	1.39 (0.87, 2.08)	−0.30 (−0.54, −0.06)	1.62 (1.26, 2.06)	1.62 (1.26, 2.09)	1.53 (1.18, 1.94)	3.46 (2.69, 4.46)	−0.42 (−0.72, −0.12)
Eastern Sub-Saharan Africa	18.81 (14.67, 24.36)	1.62 (1.23, 2.15)	17.95 (14.01, 23.20)	3.51 (2.66, 4.67)	−0.24 (−0.32, −0.16)	0.52 (0.33, 0.78)	0.45 (0.28, 0.68)	0.49 (0.31, 0.75)	0.98 (0.61, 1.48)	−0.21 (−0.30, −0.13)	1.20 (0.92, 1.51)	1.10 (0.85, 1.42)	1.14 (0.88, 1.45)	2.40 (1.84, 3.10)	−0.22 (−0.30, −0.14)
Central Sub-Saharan Africa	38.33 (30.02, 48.98)	1.13 (0.86, 1.48)	37.86 (29.51, 48.26)	2.80 (2.13, 3.69)	−0.10 (−0.20, −0.01)	1.06 (0.68, 1.56)	0.32 (0.20, 0.47)	1.05 (0.67, 1.55)	0.79 (0.49, 1.19)	−0.08 (−0.17, 0.02)	1.95 (1.52, 2.40)	0.63 (0.49, 0.80)	1.90 (1.50, 2.35)	1.51 (1.16, 1.93)	−0.15 (−0.23, −0.08)
Southern Sub-Saharan Africa	73.64 (57.38, 93.32)	2.56 (1.96, 3.27)	59.93 (47.12, 76.45)	3.98 (3.07, 5.13)	−0.73 (−1.05, −0.41)	2.05 (1.31, 3.05)	0.72 (0.45, 1.08)	1.65 (1.06, 2.44)	1.11 (0.70, 1.65)	−0.76 (−1.08, −0.43)	3.27 (2.61, 3.99)	1.29 (1.02, 1.61)	2.89 (2.28, 3.54)	1.96 (1.54, 2.44)	−0.50 (−0.74, −0.25)

ASDR, age-standardized DALY rate; DALYs, disability-adjusted life years; ASPR, age-standardized prevalence rate; ASIR, age-standardized incidence rate; No., number; UI, uncertainty interval; EAPC, estimated annual percentage change; CI, confidential interval.

### Variation in edentulism burden at the national and regional level

**Figure 2:**
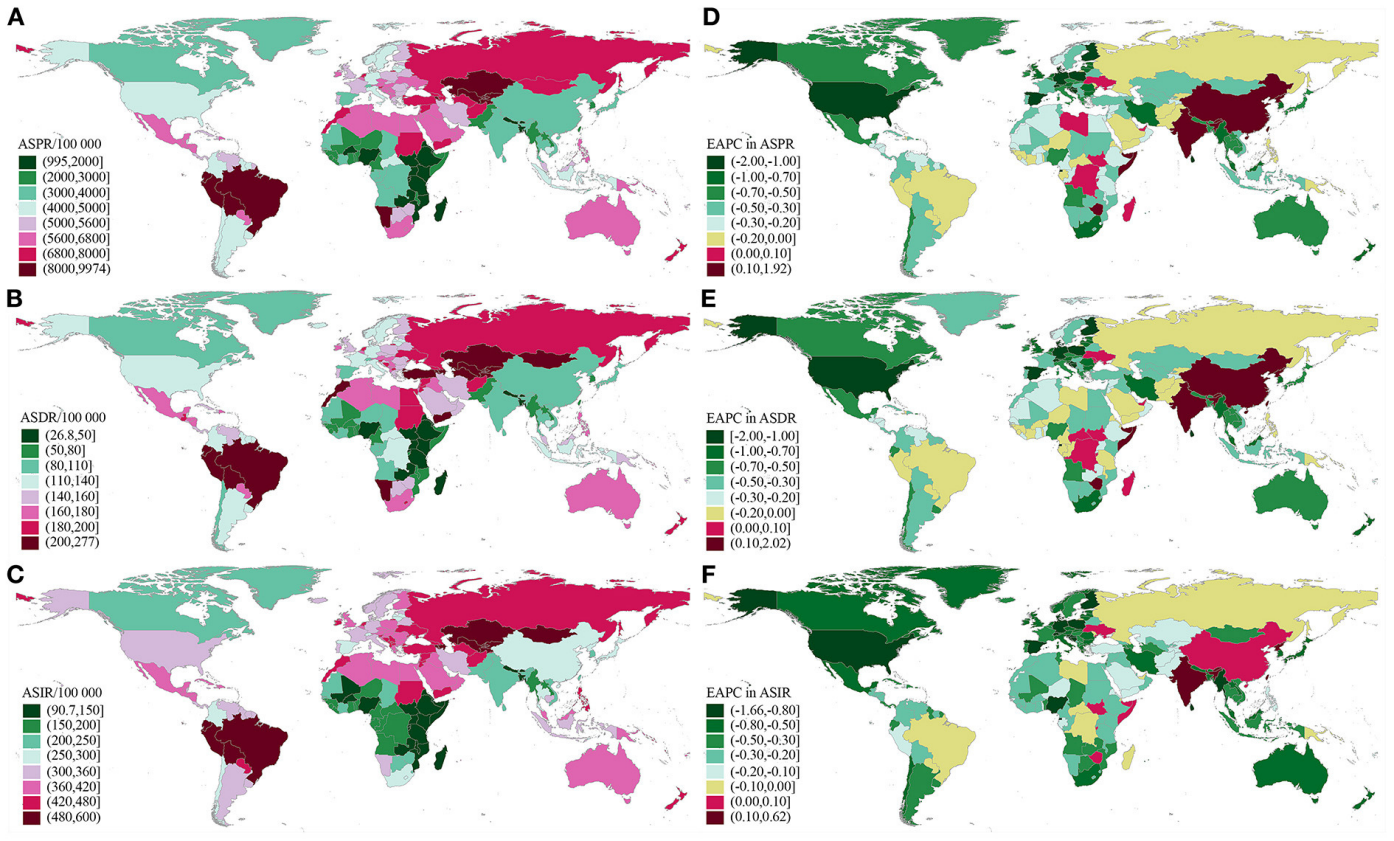
Global distribution of ASRs and the corresponding EAPCs in edentulism. **(A)** ASPR in 2019; **(B)** ASDR in 2019; **(C)** ASIR in 2019; **(D)** EAPC of ASPR from 1990 to 2019; **(E)** EAPC of ASDR from 1990 to 2019; **(F)** EAPC of ASIR from 1990 to 2019. ASRs, age-standardized rates; EAPC, estimated annual percentage change; ASPR, age-standardized prevalence rate; ASDR, age-standardized DALY rate; DALYs, disability-adjusted life years; ASIR, age-standardized incidence rates.

The global variety of ASIR, ASPR, and ASDR of edentulism was around 6.61, 10.02, and 10.28 times in 2019, respectively, with the highest ASIR, ASPR, and ASDR in Peru, and the lowest ASIR, ASPR, and ASDR in Bangladesh (Figures 2A–C, Supplementary Tables 1–3). Overall, the ASIR in 2019 was higher than 4.8/1,000 in 11 countries and territories, including Kyrgyzstan, Tajikistan, Kazakhstan, Brazil, Peru, etc., (Figure 2C, Supplementary Table 1), which also showed a severe burden in ASPR and ASDR (Figures 2A,B, Supplementary Tables 2, 3). During the period from 1990 to 2019, ASIR, ASPR, and ASDR in most countries and territories showed a downward trend, and only a very small number of countries and territories showed an upward or stable trend in ASIR, ASPR, and ASDR, such as Zimbabwe and India (Figures 2D–F, Supplementary Tables 1–3).

**Figure 3:**
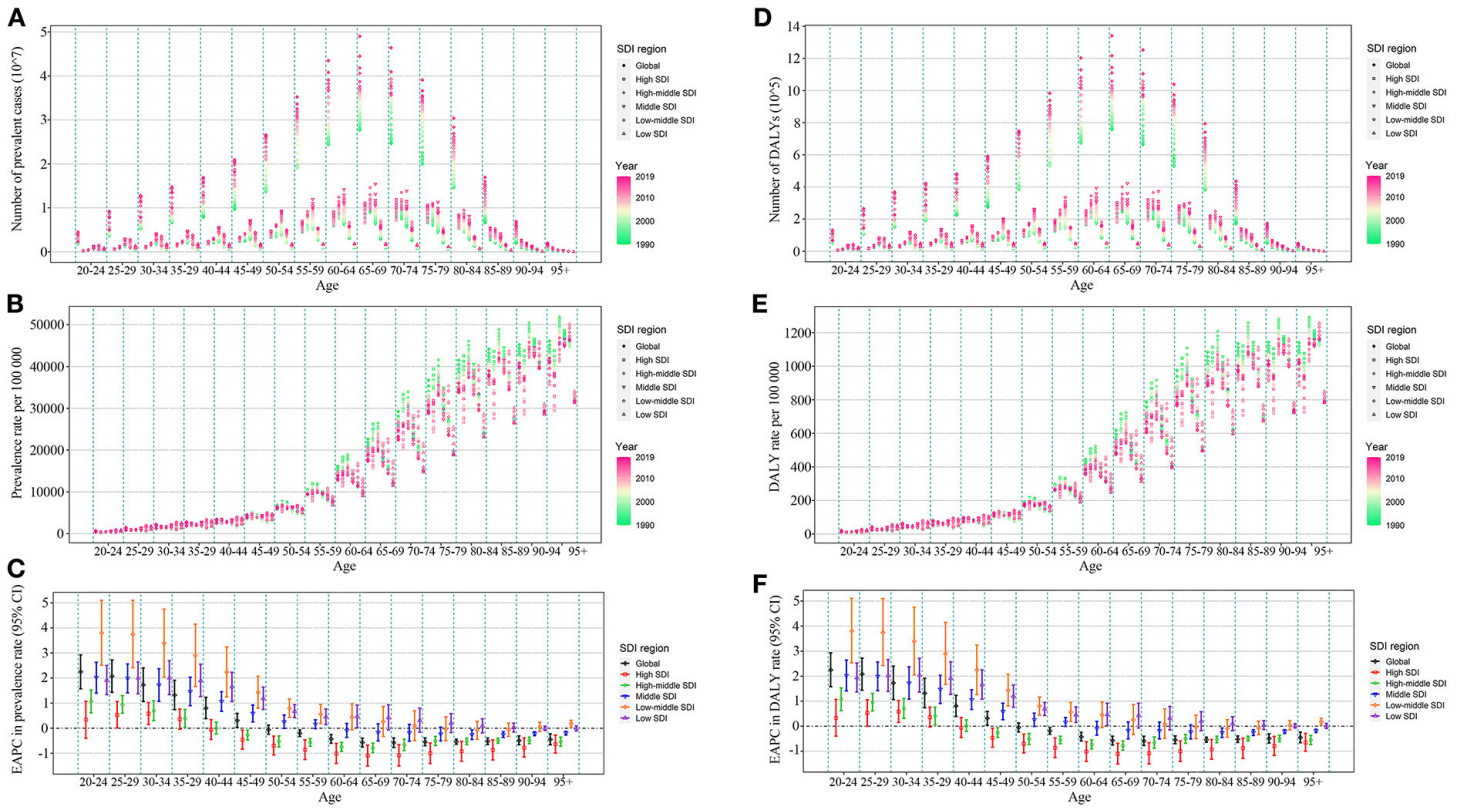
Prevalent cases, prevalence rate, EAPC of prevalence rate, DALYs, DALY rate and EAPC of DALY rate of edentulism by age group, and SDI regions from 1990 to 2019. **(A)** Prevalent cases; **(B)** prevalence rate; **(C)** EAPC of prevalence rate; **(D)** DALYs; **(E)** DALY rate; **(F)** EAPC of DALY rate. DALYs, disability-adjusted life years; SDI, socio-demographic index; EAPC, estimated annual percentage change.

Although high and high–middle SDI regions showed a decreased trend in ASIR, ASPR, and ASDR, the number of incident cases, prevalent cases, and DALYs was high in high, high–middle, and middle SDI regions and showed an increase in all SDI regions compared with 1990. Low and low–middle SDI regions showed increasing trends in ASPR and ASDR compared with 1990. Except for the ASPR and ASDR in South Asia, which showed an upward trend, the ASIR, ASPR, and ASDR in other GBD regions showed a downward or stable trend (Table 1). In almost all SDI regions, the number of incident cases, prevalent cases, and DALYs were higher in females than in males (Figure 1, Supplementary Figure 1). The number of incident cases, prevalent cases, and DALYs showed an upward trend in almost all age groups and in all SDI regions. Middle SDI region had the highest disease burden in almost all age groups (Figures 3A,D, Supplementary Figure 2A). Incidence rates began to rise in the 40–44 age group, peaked in the 70–74 age group, and began to decline after age 75. Between the ages of 40 and 74 years, the ASIRs of each SDI region tended to decrease, and after the age of 75 years, the ASIRs of each SDI region tended to increase (Supplementary Figure 2B). Prevalence rates and DALY rates started to rise at age 50 and slowed down around age 85. Prevalence rates and DALY rates in high, high–middle, and middle SDI regions tended to decline in the age group over 50 years old, while low and low–middle SDI regions tended to increase (Figures 3B,E). The incidence, prevalence, and DALY rate increased significantly in the age group <40 years old, and the EAPCs in the low–middle SDI regions were the largest (Figures 3C,F, Supplementary Figure 2C).

### The influential factors for EAPC

**Figure 4:**
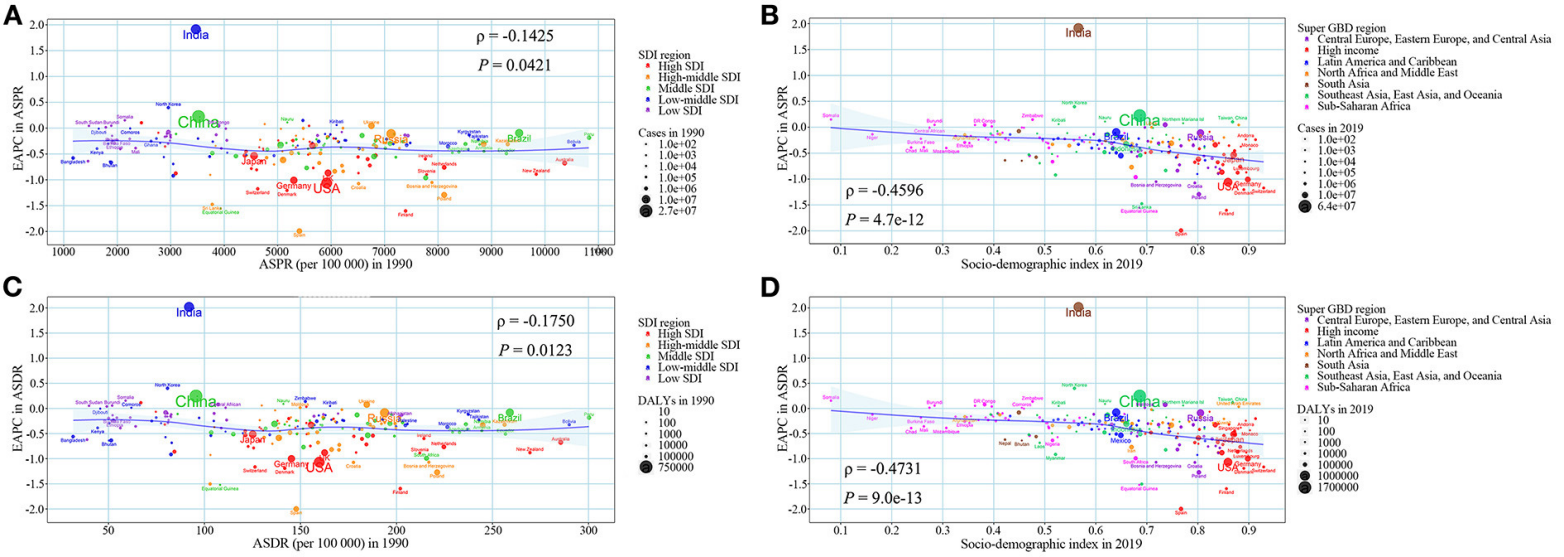
The association between ASRs in 1990, SDI in 2019, and the EAPC of ASRs from 1990 to 2019. **(A)** ASPR in 1990 and the EAPC of ASPR from 1990 to 2019; **(B)** SDI in 2019 and the EAPC of ASPR from 1990 to 2019; **(C)** ASDR in 1990 and the EAPC of ASDR from 1990 to 2019; **(D)** SDI in 2019 and the EAPC of ASDR from 1990 to 2019. The blue line was an adaptive association fitted with adaptive Loess regression based on all data points. EAPC, estimated annual percentage change; SDI, socio-demographic index; ASDR, age-standardized DALY rate; DALY, disability-adjusted life year; ASPR, age-standardized prevalence rate; ASRs, age-standardized rates.

**Figure 5:**
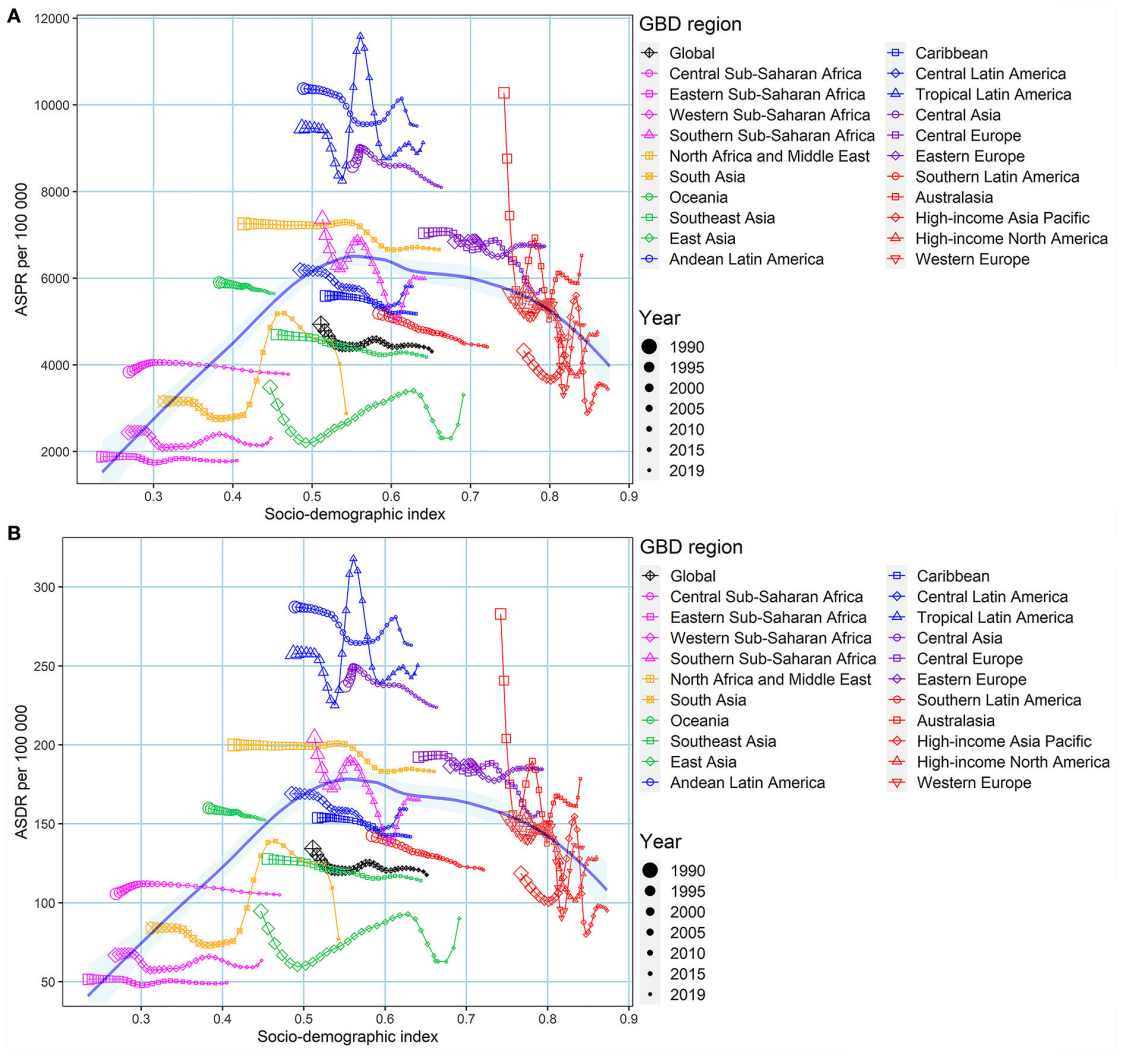
The association between ASPR, ASDR, and SDI by GBD regions from 1990 to 2019. **(A)** ASPR; **(B)** ASDR. The blue line was an adaptive association fitted with adaptive Loess regression based on all data points. GBD, global burden of disease; ASPR, age-standardized prevalence rate; DALYs, disability-adjusted life years; ASDR, age-standardized DALY rate.

We regarded ASR in 1990 as the initial burden of disease and SDI in 2019 as a proxy for socioeconomic levels and availability of healthcare services in different countries. ASPR in 1990 and ASDR in 1990 had negative correlations between the corresponding EAPCs at the national level, suggesting that edentulism burden interventions may be prioritized in countries and territories with a high disease burden of edentulism. Some countries, such as Brazil and Peru, even with a high disease burden of edentulism, appeared to have failed to take effective measures to reduce the disease burden of edentulism (Figures 4A,C). However, there was no correlation between ASIR (1990) and EAPC of ASIR from 1990 to 2019 at the national level (ρ = −0.0500, *P* = 0.4773) (Supplementary Figure 3A). The EAPCs of ASIR, ASPR, and ASDR were negatively related to SDI in 2019, suggesting that countries with better socioeconomic levels and availability of healthcare services may take corresponding measures to reduce the disease burden of edentulism (Figures 4B,D, Supplementary Figure 3B). In some GBD regions, such as Western Sub-Saharan Africa and Central Sub-Saharan Africa, ASIR, ASPR, and ASDR showed a stable or decreasing trend with increasing SDI, however, in some other GBD regions, such as Australasia, Tropical Latin America, ASIR, ASPR, and ASDR fluctuated significantly with increasing SDI, although the overall trend was declining or stable (Figure 5, Supplementary Figure 4).

## Discussion

This study provided a detailed summary of the disease burden of edentulism by sex and age groups at the global, regional, and national levels, and further analyzed temporal trends and the influencing factors over the past 30 years. Although ASIR, ASPR, and ASDR for edentulism declined over the past three decades in most countries and regions, the number of prevalent cases and DALY nearly doubled. Compared with males, females had more incident cases, prevalent cases, and DALYs, and the disease burden was heavier. Elderly people were more affected. Although ASIR, ASPR, and ASDR declined or stabilized in high, high–middle, and middle SDI regions, the disease burden in these regions was still quite high. Although the disease burden of edentulism in low and low–middle SDI regions was relatively low, the prevalence and DALY rates were on the rise. With the increase of SDI and initial burden of disease, most ASIR, ASPR, and ASDR were trending downward.

Our study found that the absolute disease burden of edentulism has still almost doubled over the past three decades, although decreasing trends were observed in ASIR, ASPR, and ASDR for edentulism at the national and global levels. Studies have shown that DALYs due to severe tooth loss decreased between 1990 and 2010 (20), which suggested that the temporal trend and influencing factors of edentulism have already changed during the past last decade. One possible reason for the doubling of the absolute disease burden is population aging. A study showed that between 1990 and 2017, the number of people aged 65 and above increased from 327.6 million in 1990 to 673.7 million in 2017, and the proportion of the population increased from 6.1 to 8.8% (32). The increase in the number of people aged 65 and over will inevitably lead to an increase in the number of edentulism cases and disease burden. Another potential factor could be change in dietary patterns. A study had shown that the type and number of ultra-processed foods and beverages in the world's food supply were increasing substantially (33). Added sugars in beverages can lead to tooth decay. Studies have shown that eating sweets can lead to an increase in the incidence of dental caries, with an *OR*-value of 2.4 (34), and dental caries was one of the reasons for tooth loss (3).

The relationship between gender and edentulism was inconsistent. Some studies were showing that the burden of total tooth loss was similar among women and men or that more males were affected than females (1, 35). However, there were also studies showing that the disease burden was higher in women than in men (15, 36, 37). Our study found that in almost all SDI regions, the number of incident cases, prevalent cases, and DALYs were higher in females than in males. The Biological causes of more tooth loss in women may be estrogen deficiency and osteoporosis. Estrogen deficiency in women due to various reasons, such as menopause, ovarian disease, etc., can lead to osteoporosis, and osteoporosis is closely related to periodontitis, which is one of the causes of tooth loss (38–41). In terms of social factors of edentulism, females may be more concerned about their appearance and dental condition than males, and this may be more pronounced among females of high socioeconomic status. Therefore, females may receive complete denture treatment more often than males (2, 42).

Our study shows that the prevalence of edentulism increases exponentially with age, which is consistent with the findings of a previous study (1). Moreover, our results found that prevalence rates and DALY rates in low and low–middle SDI regions tended to increase in the age group over 50 years old, while high, high–middle, and middle SDI regions tended to decline. Edentulism is the ultimate marker of the common oral health disease burden (2). Its development has gone through a long process. Poor oral hygiene habits at a young age, periodontitis, dental caries, and other factors, experienced over time, may lead to edentulism in old age (39). Thus, in economically less developed regions, active treatment and prevention of oral diseases are necessary.

Our study showed a trend of higher disease burden of edentulism in more economically developed regions. In 2019, the number of prevalent cases in high SDI, high–middle SDI, and middle SDI regions was 8.11, 9.63, and 10.95 million, respectively, significantly higher than the 4.78 and 1.72 million in regions with low–middle SDI, low SDI. However, we also found that in regions with a better economy, ASIR, ASPR, and ASDR tended to show a declining or stable trend, while regions with a poor economy showed an upward trend. The relationship between socioeconomics and the disease burden of edentulism is complex. On the one hand, as many studies have found, higher socioeconomic status is a protective factor for edentulism (15, 35, 43). On the other hand, economic improvement at the national level may increase the type and quantity of ultra–processed foods and various types of beverages, increase the incidence of dental caries and periodontitis, and thus increase the disease burden of edentulism (33). In addition, in some cultures, females, especially females with higher socioeconomic status, may choose dentures because they are concerned for their appearance and the appearance of their teeth, which also increases the prevalence of edentulism in regions with better economic levels to a certain extent.

Although some countries with higher SDI or higher initial disease burden had larger ASIR, ASPR, and ASDR decline, the current and future disease burden of edentulism is still very high. As a common disease of the elderly, the disease burden of edentulism may further increase in today's increasingly aging world (44). In our analysis, we found a high disease burden of edentulism in parts of South America and an increasing trend in China and India. Tooth extraction seems to be a common trend in India and South America, and many people, especially the elderly, believe that tooth loss is a natural part of aging (45, 46). China is currently facing a rapid rural-to-urban migration, which will affect dietary and oral hygiene practices, and further affect the disease burden of edentulism (47, 48). The diagnostic rate and reporting rate of edentulism in areas with low economic levels may be lower than those in areas with high economic levels, which is also one of the potential reasons for low disease burden in areas with low economic levels, and one of the possible reasons for the rising trend of the disease burden in low economic areas. At present, COVID-19 is spreading all over the world. Most economies are affected by COVID-19, and the future recovery is uncertain. These countries and regions have large populations, and their current disease status and trends suggest that the burden of edentulism may still increase further in the future. Dental caries and periodontal disease are the leading causes of tooth loss, and both are preventable (3). Treatment and prevention of dental caries and periodontal disease are needed in various countries to reduce the disease burden caused by edentulism.

Our research has some advantages. First, the data used in our study is based on all currently available data that use powerful computational methods, and its quality is optimal. Second, our research is the latest detailed description of the geographic and temporal trends of global edentulism and its influencing factors, which can provide a basis and reference for mitigating the burden of edentulism.

Our study also has some limitations. First, since the data of the GBD database is estimated using a mathematical model based on a large amount of data, there may be a certain deviation from the actual data. Second, although the GBD researcher uses almost all available data, such as vital registration, and oral autopsy, there is still no data in some countries. Using data from neighboring countries to estimate the disease burden of these countries will result in inevitable bias. Third, there are differences in the quality and quantity of edentulism data sources in countries with different economic conditions, as well as differences in the importance attached to edentulism among different countries, which may lead to heterogeneity among different countries. Finally, for some countries with small populations, where small changes in the number of incident cases or prevalent cases can lead to large changes in ASIR, ASPR, and ASDR, estimates in these countries may be biased (21, 22, 26, 27).

## Conclusion

In summary, during the past three decades, although ASIR, ASPR, and ASDR of edentulism have declined or stabilized in most countries and regions, the absolute disease burden of edentulism has nearly doubled. Although the increasing trend of the ASIR, ASPR, and ASDR of edentulism in high SDI, high–middle SDI, and middle SDI regions was not obvious, the disease burden was still high. Although the absolute disease burden of edentulism was lower in low SDI and low–middle SDI regions, ASPR and ASDR were on the rise in those regions. Countries around the world should actively take a series of measures including the prevention and treatment of dental caries and periodontal disease to reduce the disease burden of edentulism.

## Data availability statement

Publicly available datasets were analyzed in this study. This data can be found at: http://ghdx.healthdata.org/gbd-results-tool.

## Ethics statement

The studies involving human participants were reviewed and approved by Institutional Review Boards of Qilu Hospital of Shandong University. Written informed consent for participation was not required for this study in accordance with the national legislation and the institutional requirements.

## Author contributions

XL: formal analysis, data curation, and writing-original draft preparation. JM and HC: data curation, visualization, and results interpretation. XY: conceptualization, supervision, results interpretation, writing-reviewing and editing, and funding acquisition. All authors contributed to the article and approved the submitted version.

## Funding

This work was supported by the National Natural Science Foundation of China (82103912), the China Post-doctoral Science Foundation (2021M700080), and the Shandong Provincial Natural Science Foundation (ZR2020QH302). The funders were not involved in the collection, analysis, or interpretation of data, or the writing or submitting of this report.

## Conflict of interest

The authors declare that the research was conducted in the absence of any commercial or financial relationships that could be construed as a potential conflict of interest.

## Publisher's note

All claims expressed in this article are solely those of the authors and do not necessarily represent those of their affiliated organizations, or those of the publisher, the editors and the reviewers. Any product that may be evaluated in this article, or claim that may be made by its manufacturer, is not guaranteed or endorsed by the publisher.

## Abbreviations

GBD: global burden of disease
ASIR: age-standardized incidence rate
ASPR: age-standardized prevalence rate
DALYs: disability-adjusted life years
ASDR: age-standardized DALY rate
GHDx: Global Health Data Exchange
SDI: socio-demographic index
ASRs: age-standardized rates
UI: uncertainty interval
EAPC: estimated annual percentage change
CI: confidence interval.
