# Supplementary material for: Spatiotemporal trends of disease burden of edentulism from 1990 to 2019: A global, regional, and national analysis
Source: Front Public Health. 2022 Nov 28;10:940355. doi: 10.3389/fpubh.2022.940355 (PMC9742533; doi:10.3389/fpubh.2022.940355)
Supplement: Supplementary file 1 [file Data_Sheet_1.PDF]

Supplementary Material

1 Supplementary Figures and Tables

1.1 Supplementary Tables

Supplementary table 1. Incidence and age-standardized incidence rate per 1000 people for edentulism in 1990 and 2019, and its estimated annual percentage change in 204 countries and territories from 1990 to 2019.

	1990		2019		EAPC of ASIR (95%CI)
	ASIR/1000 (95%UI)	Incident cases*10^5 (95%UI)	ASIR/1000 (95%UI)	Incident cases*10^5 (95%UI)	From 1990 to 2019
Afghanistan	4.59(3.66,5.63)	0.35(0.28,0.45)	4.52(3.60,5.51)	0.80(0.63,1.01)	-0.14(-0.22,-0.07)
Albania	4.68(3.76,5.67)	0.11(0.09,0.13)	4.22(3.35,5.15)	0.17(0.13,0.21)	-0.46(-0.51,-0.42)
Algeria	4.30(3.42,5.22)	0.63(0.50,0.77)	4.12(3.27,5.10)	1.57(1.24,1.96)	-0.21(-0.24,-0.19)
American Samoa	3.82(3.01,4.67)	0.00(0.00,0.00)	3.80(2.96,4.66)	0.00(0.00,0.00)	-0.03(-0.04,-0.01)
Andorra	3.49(2.70,4.34)	0.00(0.00,0.00)	3.28(2.53,4.13)	0.00(0.00,0.01)	-0.24(-0.27,-0.20)
Angola	1.90(1.49,2.34)	0.11(0.09,0.14)	1.72(1.35,2.12)	0.29(0.22,0.37)	-0.50(-0.56,-0.43)
Antigua and Barbuda	3.57(2.81,4.46)	0.00(0.00,0.00)	3.28(2.53,4.11)	0.00(0.00,0.00)	-0.31(-0.32,-0.29)
Argentina	3.74(2.94,4.60)	1.22(0.95,1.51)	3.28(2.56,4.07)	1.70(1.33,2.11)	-0.43(-0.46,-0.40)
Armenia	5.42(4.39,6.46)	0.17(0.13,0.21)	5.12(4.11,6.14)	0.21(0.16,0.26)	-0.34(-0.45,-0.24)
Australia	5.69(5.38,5.98)	1.07(1.01,1.12)	4.14(3.25,5.09)	1.49(1.15,1.82)	-0.71(-1.15,-0.27)
Austria	4.41(3.47,5.35)	0.47(0.37,0.56)	3.99(3.12,4.89)	0.59(0.47,0.73)	-0.69(-1.07,-0.30)
Azerbaijan	5.21(4.24,6.22)	0.30(0.24,0.37)	4.94(3.98,5.96)	0.55(0.42,0.70)	-0.35(-0.49,-0.21)
Bahamas	3.37(2.61,4.23)	0.01(0.00,0.01)	3.21(2.48,4.04)	0.01(0.01,0.02)	-0.19(-0.21,-0.17)
Bahrain	3.94(3.12,4.88)	0.01(0.01,0.01)	3.79(2.97,4.73)	0.06(0.04,0.07)	-0.17(-0.19,-0.15)
Bangladesh	1.06(0.82,1.34)	0.49(0.38,0.62)	0.91(0.70,1.15)	1.16(0.88,1.47)	-0.55(-0.58,-0.52)
Barbados	3.58(2.77,4.42)	0.01(0.01,0.01)	3.37(2.62,4.24)	0.02(0.01,0.02)	-0.24(-0.26,-0.22)
Belarus	3.75(2.95,4.65)	0.49(0.38,0.62)	3.53(2.78,4.34)	0.53(0.40,0.66)	-0.28(-0.34,-0.23)
Belgium	4.45(3.53,5.40)	0.63(0.49,0.75)	4.02(3.15,4.93)	0.75(0.59,0.91)	-0.47(-0.62,-0.33)
Belize	3.81(2.96,4.73)	0.00(0.00,0.00)	3.53(2.74,4.44)	0.01(0.01,0.01)	-0.27(-0.30,-0.25)
Benin	1.89(1.46,2.39)	0.04(0.03,0.06)	1.76(1.37,2.25)	0.11(0.08,0.14)	-0.32(-0.36,-0.28)
Bermuda	3.34(2.58,4.18)	0.00(0.00,0.00)	3.08(2.37,3.86)	0.00(0.00,0.00)	-0.32(-0.34,-0.30)
Bhutan	1.58(1.24,1.98)	0.00(0.00,0.00)	1.33(1.04,1.67)	0.01(0.01,0.01)	-0.60(-0.61,-0.59)
Bolivia	6.27(5.24,7.25)	0.24(0.20,0.28)	5.85(4.83,6.89)	0.57(0.47,0.69)	-0.25(-0.26,-0.24)
Bosnia and Herzegovina	4.99(4.02,6.01)	0.23(0.18,0.29)	4.41(3.50,5.36)	0.25(0.19,0.30)	-0.97(-1.55,-0.40)
Botswana	2.67(2.11,3.33)	0.02(0.02,0.03)	2.43(1.92,3.02)	0.05(0.04,0.06)	-0.31(-0.32,-0.31)
Brazil	6.06(5.08,6.97)	6.15(5.12,7.20)	5.88(4.88,6.81)	14.5(11.97,16.97)	-0.06(-0.26,0.15)
Brunei	2.16(1.68,2.70)	0.00(0.00,0.00)	2.14(1.65,2.65)	0.01(0.01,0.01)	-0.09(-0.10,-0.08)
Bulgaria	4.19(3.31,5.11)	0.52(0.40,0.64)	3.75(2.93,4.64)	0.47(0.36,0.58)	-0.70(-0.97,-0.43)
Burkina Faso	1.36(1.05,1.74)	0.07(0.05,0.08)	1.25(0.96,1.60)	0.13(0.10,0.17)	-0.25(-0.45,-0.06)
Burundi	1.17(0.90,1.49)	0.03(0.03,0.04)	1.19(0.92,1.51)	0.07(0.05,0.09)	0.05(0.01,0.08)
Cape Verde	1.84(1.44,2.35)	0.00(0.00,0.01)	1.63(1.28,2.09)	0.01(0.01,0.01)	-0.52(-0.57,-0.48)
Cambodia	3.45(2.70,4.27)	0.17(0.14,0.22)	3.02(2.35,3.76)	0.39(0.30,0.50)	-0.47(-0.53,-0.42)
Cameroon	1.77(1.37,2.23)	0.09(0.07,0.12)	1.69(1.33,2.16)	0.25(0.20,0.32)	-0.24(-0.30,-0.18)
Canada	2.79(2.17,3.44)	0.88(0.69,1.08)	2.22(1.74,2.80)	1.26(0.97,1.62)	-0.57(-0.83,-0.31)
Central African Republic	2.01(1.59,2.50)	0.03(0.03,0.04)	1.99(1.57,2.48)	0.06(0.05,0.08)	-0.09(-0.12,-0.07)
Chad	2.37(1.83,3.01)	0.08(0.06,0.10)	2.17(1.68,2.77)	0.16(0.12,0.20)	-0.40(-0.52,-0.28)

Chile	3.48(2.72,4.33)	0.36(0.28,0.46)	2.96(2.29,3.70)	0.71(0.55,0.89)	-0.51(-0.54,-0.49)
China	2.85(2.24,3.52)	24.66(18.94,31.01)	2.72(2.14,3.38)	55.32(42.43,69.92)	0.01(-0.53,0.56)
Colombia	3.20(2.49,3.99)	0.65(0.51,0.81)	2.88(2.25,3.60)	1.53(1.20,1.93)	-0.25(-0.33,-0.16)
Comoros	1.35(1.05,1.72)	0.00(0.00,0.00)	1.34(1.04,1.71)	0.01(0.01,0.01)	-0.05(-0.15,0.06)
Congo	1.81(1.43,2.25)	0.03(0.02,0.03)	1.73(1.36,2.15)	0.06(0.05,0.08)	-0.27(-0.58,0.05)
Cook Islands	3.95(3.12,4.81)	0.00(0.00,0.00)	3.69(2.86,4.54)	0.00(0.00,0.00)	-0.21(-0.22,-0.20)
Costa Rica	3.76(2.98,4.64)	0.07(0.06,0.09)	3.45(2.69,4.29)	0.18(0.14,0.23)	-0.32(-0.33,-0.31)
Croatia	4.35(3.43,5.30)	0.28(0.22,0.35)	3.91(3.08,4.84)	0.29(0.23,0.36)	-1.01(-1.58,-0.43)
Cuba	3.99(3.13,4.96)	0.41(0.32,0.50)	3.66(3.30,4.07)	0.66(0.60,0.74)	-0.36(-0.42,-0.30)
Cyprus	3.86(3.02,4.75)	0.03(0.02,0.04)	3.55(2.76,4.44)	0.07(0.05,0.08)	-0.30(-0.33,-0.27)
Czech	4.43(3.51,5.43)	0.58(0.45,0.70)	3.94(3.11,4.89)	0.72(0.55,0.88)	-0.61(-0.84,-0.38)
Cote d'Ivoire	2.32(1.82,2.94)	0.13(0.10,0.16)	2.24(1.75,2.84)	0.31(0.24,0.40)	-0.21(-0.67,0.25)
North Korea	2.45(1.89,3.08)	0.38(0.29,0.49)	2.68(2.09,3.31)	0.86(0.67,1.07)	0.31(0.29,0.34)
DR Congo	1.97(1.54,2.43)	0.44(0.34,0.56)	1.97(1.55,2.45)	1.06(0.82,1.36)	-0.04(-0.13,0.06)
Denmark	3.59(2.79,4.46)	0.26(0.20,0.32)	3.12(2.41,3.90)	0.31(0.24,0.38)	-1.15(-2.13,-0.17)
Djibouti	1.06(0.81,1.34)	0.00(0.00,0.00)	1.05(0.81,1.34)	0.01(0.01,0.01)	-0.02(-0.07,0.03)
Dominica	3.81(2.98,4.71)	0.00(0.00,0.00)	3.41(2.63,4.25)	0.00(0.00,0.00)	-0.4(-0.43,-0.37)
Dominican Republic	4.48(3.55,5.43)	0.18(0.15,0.23)	4.04(3.19,4.97)	0.39(0.31,0.49)	-0.37(-0.49,-0.24)
Ecuador	5.77(4.69,6.79)	0.35(0.29,0.42)	5.38(4.40,6.35)	0.86(0.70,1.03)	-0.43(-0.71,-0.14)
Egypt	4.45(3.55,5.44)	1.56(1.25,1.94)	4.15(3.29,5.08)	3.16(2.48,3.98)	-0.27(-0.28,-0.26)
El Salvador	3.92(3.08,4.84)	0.13(0.10,0.16)	3.65(2.87,4.53)	0.21(0.17,0.26)	-0.26(-0.26,-0.25)
Equatorial Guinea	1.97(1.54,2.44)	0.00(0.00,0.01)	1.51(1.18,1.88)	0.01(0.01,0.02)	-1.22(-1.37,-1.07)
Eritrea	1.17(0.90,1.49)	0.02(0.01,0.02)	1.11(0.85,1.41)	0.04(0.03,0.05)	-0.14(-0.19,-0.09)
Estonia	3.72(2.93,4.58)	0.07(0.06,0.09)	3.46(2.69,4.29)	0.08(0.06,0.09)	-0.72(-1.12,-0.32)
Eswatini	2.50(1.96,3.09)	0.01(0.01,0.01)	2.32(1.84,2.86)	0.02(0.02,0.02)	-0.27(-0.52,-0.02)
Ethiopia	1.28(0.99,1.62)	0.31(0.24,0.40)	1.19(0.92,1.51)	0.64(0.49,0.82)	-0.27(-0.47,-0.08)
Fiji	4.20(3.32,5.14)	0.02(0.01,0.02)	3.96(3.10,4.88)	0.03(0.02,0.04)	-0.18(-0.18,-0.17)
Finland	4.97(4.29,5.72)	0.33(0.29,0.37)	3.64(2.84,4.53)	0.36(0.28,0.45)	-1.54(-2.18,-0.90)
France	3.84(2.98,4.71)	2.88(2.23,3.52)	3.47(2.69,4.30)	3.83(2.94,4.69)	-0.31(-1.08,0.47)
Gabon	1.65(1.29,2.06)	0.01(0.01,0.01)	1.58(1.24,1.95)	0.02(0.02,0.03)	-0.18(-0.19,-0.16)
Gambia	1.86(1.44,2.34)	0.01(0.01,0.01)	1.80(1.40,2.28)	0.02(0.02,0.03)	-0.17(-0.22,-0.13)
Georgia	4.64(3.71,5.64)	0.30(0.23,0.37)	4.56(3.63,5.54)	0.25(0.20,0.30)	-0.22(-0.35,-0.09)
Germany	3.62(2.80,4.50)	4.24(3.29,5.27)	3.20(2.49,4.01)	4.99(3.82,6.20)	-1.07(-1.71,-0.43)
Ghana	1.71(1.34,2.18)	0.13(0.10,0.17)	1.56(1.21,2.00)	0.30(0.23,0.38)	-0.24(-0.50,0.02)
Greece	3.91(3.07,4.82)	0.57(0.44,0.71)	3.60(2.82,4.46)	0.66(0.52,0.81)	-0.55(-0.97,-0.13)
Greenland	2.83(2.22,3.58)	0.00(0.00,0.00)	2.39(1.87,3.01)	0.00(0.00,0.00)	-0.59(-0.62,-0.56)
Grenada	3.80(2.97,4.70)	0.00(0.00,0.00)	3.37(2.62,4.23)	0.00(0.00,0.01)	-0.44(-0.47,-0.42)
Guam	3.62(2.83,4.43)	0.00(0.00,0.00)	3.52(2.73,4.35)	0.01(0.01,0.01)	-0.40(-0.72,-0.09)
Guatemala	4.38(3.51,5.35)	0.19(0.16,0.24)	4.14(3.30,5.05)	0.51(0.41,0.62)	-0.23(-0.30,-0.15)
Guinea	1.85(1.44,2.37)	0.07(0.05,0.09)	1.76(1.38,2.24)	0.11(0.09,0.15)	-0.21(-0.26,-0.16)
Guinea-Bissau	1.90(1.48,2.42)	0.01(0.01,0.01)	1.81(1.41,2.30)	0.02(0.01,0.02)	-0.20(-0.23,-0.16)
Guyana	3.92(3.07,4.83)	0.02(0.01,0.02)	3.56(2.76,4.42)	0.02(0.02,0.03)	-0.35(-0.36,-0.35)
Haiti	4.00(3.11,4.92)	0.15(0.11,0.19)	3.89(3.02,4.88)	0.31(0.24,0.40)	-0.12(-0.13,-0.11)
Honduras	4.00(3.16,4.92)	0.10(0.08,0.12)	3.77(2.95,4.69)	0.26(0.21,0.33)	-0.22(-0.23,-0.22)
Hungary	4.54(3.60,5.54)	0.64(0.50,0.78)	4.04(3.18,5.00)	0.68(0.52,0.83)	-0.38(-0.61,-0.16)
Iceland	3.79(2.94,4.67)	0.01(0.01,0.01)	3.38(2.60,4.20)	0.02(0.01,0.02)	-0.44(-0.47,-0.42)
India	2.67(2.08,3.36)	12.59(9.54,16.24)	2.42(1.88,3.02)	27.96(21.65,35.33)	0.61(-0.11,1.34)
Indonesia	3.59(2.84,4.40)	3.97(3.12,5.04)	3.24(2.55,3.99)	7.62(5.87,9.70)	-0.31(-0.35,-0.27)
Iran	3.70(2.95,4.56)	1.16(0.91,1.47)	3.52(2.80,4.34)	2.87(2.26,3.58)	-0.66(-1.14,-0.19)
Iraq	4.30(3.41,5.31)	0.39(0.31,0.49)	4.10(3.25,5.08)	1.18(0.94,1.50)	-0.29(-0.34,-0.24)

Ireland	4.88(3.95,5.80)	0.19(0.15,0.22)	4.20(3.28,5.13)	0.29(0.22,0.35)	-0.55(-0.76,-0.34)
Israel	4.35(3.43,5.31)	0.20(0.16,0.24)	3.90(3.05,4.79)	0.41(0.32,0.50)	-0.37(-0.42,-0.33)
Italy	3.56(2.79,4.38)	2.97(2.31,3.66)	3.42(2.67,4.24)	3.88(3.06,4.77)	-0.63(-1.17,-0.08)
Jamaica	3.78(2.94,4.66)	0.06(0.05,0.08)	3.56(2.78,4.42)	0.11(0.08,0.13)	-0.20(-0.21,-0.18)
Japan	3.42(2.74,4.12)	5.77(4.55,7.04)	2.82(2.24,3.46)	8.17(6.37,10.05)	-0.60(-1.33,0.13)
Jordan	4.42(3.51,5.37)	0.08(0.06,0.10)	4.20(3.32,5.18)	0.35(0.28,0.45)	-0.27(-0.30,-0.24)
Kazakhstan	5.59(4.60,6.61)	0.78(0.63,0.94)	5.30(4.30,6.33)	1.01(0.79,1.24)	-0.16(-0.41,0.09)
Kenya	1.08(0.84,1.37)	0.11(0.09,0.14)	1.07(0.83,1.36)	0.30(0.23,0.39)	-0.24(-0.34,-0.15)
Kiribati	4.38(3.48,5.30)	0.00(0.00,0.00)	4.41(3.50,5.35)	0.00(0.00,0.00)	0.03(0.02,0.05)
Kuwait	3.94(3.09,4.83)	0.04(0.03,0.05)	3.74(2.95,4.64)	0.14(0.11,0.18)	-0.22(-0.25,-0.19)
Kyrgyzstan	5.29(4.29,6.28)	0.17(0.14,0.21)	5.25(4.29,6.26)	0.28(0.22,0.35)	-0.11(-0.21,-0.01)
Laos	2.34(1.81,2.97)	0.05(0.04,0.07)	1.97(1.52,2.49)	0.09(0.07,0.12)	-0.63(-1.24,-0.02)
Latvia	3.09(2.41,3.87)	0.11(0.08,0.14)	2.95(2.30,3.71)	0.10(0.08,0.13)	-0.77(-1.32,-0.21)
Lebanon	4.38(3.48,5.32)	0.11(0.09,0.14)	4.12(3.25,5.12)	0.22(0.17,0.27)	-0.27(-0.28,-0.25)
Lesotho	2.95(2.33,3.64)	0.04(0.03,0.05)	2.73(2.16,3.38)	0.05(0.04,0.06)	-0.26(-0.27,-0.24)
Liberia	1.90(1.47,2.42)	0.02(0.02,0.03)	1.87(1.45,2.39)	0.05(0.04,0.06)	-0.26(-0.37,-0.15)
Libya	4.02(3.16,4.95)	0.09(0.07,0.11)	4.08(3.23,5.03)	0.25(0.20,0.31)	-0.05(-0.10,0.01)
Lithuania	3.15(2.45,3.91)	0.14(0.11,0.18)	2.90(2.25,3.67)	0.14(0.11,0.18)	-0.79(-1.22,-0.35)
Luxembourg	4.30(3.40,5.25)	0.02(0.02,0.03)	3.79(2.93,4.69)	0.03(0.03,0.04)	-0.84(-1.43,-0.25)
Madagascar	1.13(0.86,1.43)	0.07(0.05,0.09)	1.12(0.86,1.44)	0.16(0.12,0.21)	-0.01(-0.04,0.01)
Malawi	1.36(1.05,1.72)	0.07(0.05,0.09)	1.29(0.99,1.64)	0.12(0.09,0.16)	-0.23(-0.57,0.11)
Malaysia	4.16(3.30,5.10)	0.44(0.35,0.55)	3.68(2.91,4.55)	1.07(0.83,1.34)	-0.77(-1.44,-0.09)
Maldives	3.00(2.32,3.77)	0.00(0.00,0.00)	2.62(2.02,3.28)	0.01(0.01,0.01)	-0.48(-0.52,-0.44)
Mali	1.56(1.21,2.01)	0.07(0.06,0.09)	1.43(1.10,1.82)	0.14(0.11,0.19)	-0.39(-0.83,0.05)
Malta	3.44(2.65,4.32)	0.01(0.01,0.02)	2.94(2.25,3.72)	0.02(0.02,0.03)	-0.67(-0.82,-0.53)
Marshall Islands	4.28(3.40,5.18)	0.00(0.00,0.00)	4.13(3.23,5.06)	0.00(0.00,0.00)	-0.09(-0.11,-0.08)
Mauritania	2.30(1.79,2.91)	0.03(0.02,0.03)	2.17(1.69,2.75)	0.05(0.04,0.07)	-0.27(-0.43,-0.11)
Mauritius	4.27(3.39,5.23)	0.04(0.03,0.04)	3.83(3.02,4.68)	0.07(0.05,0.09)	-0.34(-0.76,0.09)
Mexico	4.34(3.47,5.31)	2.17(1.74,2.68)	4.14(3.31,5.09)	5.14(4.06,6.42)	-0.57(-0.83,-0.31)
Micronesia	4.33(3.42,5.23)	0.00(0.00,0.00)	4.17(3.28,5.13)	0.00(0.00,0.00)	-0.13(-0.13,-0.13)
Monaco	3.33(2.58,4.15)	0.00(0.00,0.00)	2.97(2.28,3.75)	0.00(0.00,0.00)	-0.38(-0.39,-0.37)
Mongolia	5.30(4.28,6.32)	0.06(0.05,0.08)	4.94(3.98,5.97)	0.14(0.11,0.18)	-0.33(-0.42,-0.24)
Montenegro	4.49(3.57,5.50)	0.03(0.02,0.04)	4.24(3.36,5.20)	0.04(0.03,0.05)	-0.30(-0.36,-0.24)
Morocco	4.96(3.98,6.00)	0.81(0.65,1.00)	4.59(3.69,5.57)	1.62(1.28,2.01)	-0.30(-0.35,-0.24)
Mozambique	1.26(0.98,1.61)	0.09(0.07,0.12)	1.15(0.89,1.47)	0.16(0.13,0.21)	-0.36(-0.38,-0.35)
Myanmar	2.33(1.79,2.96)	0.57(0.44,0.74)	1.90(1.47,2.42)	0.91(0.70,1.17)	-0.82(-1.59,-0.05)
Namibia	3.38(2.67,4.14)	0.04(0.03,0.04)	3.13(2.48,3.86)	0.06(0.05,0.08)	-0.28(-0.35,-0.21)
Nauru	3.87(3.03,4.74)	0.00(0.00,0.00)	3.90(3.05,4.81)	0.00(0.00,0.00)	0.03(-0.08,0.15)
Nepal	1.27(1.00,1.61)	0.11(0.09,0.14)	1.07(0.82,1.35)	0.23(0.17,0.29)	-0.60(-0.77,-0.44)
Netherlands	5.08(4.11,6.05)	0.94(0.76,1.10)	4.58(3.64,5.54)	1.33(1.05,1.58)	-0.69(-1.10,-0.29)
New Zealand	5.73(4.65,6.92)	0.21(0.17,0.25)	4.65(3.66,5.76)	0.31(0.24,0.38)	-0.70(-1.01,-0.39)
Nicaragua	3.96(3.14,4.86)	0.07(0.06,0.09)	3.75(2.95,4.62)	0.19(0.15,0.24)	-0.23(-0.24,-0.21)
Niger	1.90(1.48,2.43)	0.07(0.05,0.08)	1.84(1.44,2.36)	0.18(0.14,0.23)	-0.17(-0.23,-0.11)
Nigeria	1.34(1.04,1.71)	0.65(0.50,0.83)	1.24(0.95,1.57)	1.26(0.97,1.63)	-0.85(-1.71,0.03)
Niue	4.05(3.17,4.94)	0.00(0.00,0.00)	3.87(3.01,4.72)	0.00(0.00,0.00)	-0.18(-0.19,-0.16)
North Macedonia	4.75(3.78,5.74)	0.10(0.07,0.12)	4.39(3.48,5.36)	0.14(0.11,0.17)	-0.37(-0.42,-0.31)
Northern Mariana Islands	3.66(2.84,4.48)	0.00(0.00,0.00)	3.66(2.85,4.49)	0.00(0.00,0.00)	0.03(0.01,0.06)
Norway	3.66(2.87,4.51)	0.22(0.17,0.26)	3.44(2.69,4.25)	0.29(0.22,0.36)	-0.51(-1.92,0.92)
Oman	3.99(3.15,4.91)	0.04(0.03,0.05)	3.78(2.98,4.67)	0.12(0.09,0.15)	-0.23(-0.25,-0.21)

Pakistan	2.34(1.86,2.91)	1.39(1.10,1.73)	2.24(1.77,2.80)	2.61(2.05,3.27)	-0.12(-0.74,0.51)
Palau	3.97(3.11,4.90)	0.00(0.00,0.00)	3.82(2.99,4.67)	0.00(0.00,0.00)	-0.12(-0.13,-0.10)
Palestine	4.61(3.71,5.58)	0.05(0.04,0.06)	4.33(3.42,5.30)	0.13(0.10,0.17)	-0.22(-0.24,-0.21)
Panama	3.73(2.94,4.64)	0.06(0.05,0.08)	3.33(2.63,4.21)	0.14(0.11,0.18)	-0.39(-0.41,-0.37)
Papua New Guinea	4.33(3.44,5.28)	0.09(0.07,0.11)	4.19(3.29,5.13)	0.22(0.17,0.28)	-0.09(-0.12,-0.07)
Paraguay	4.88(3.95,5.79)	0.12(0.09,0.14)	4.41(3.51,5.32)	0.26(0.21,0.32)	-0.07(-0.69,0.56)
Peru	6.39(5.42,7.33)	0.88(0.75,1.03)	6.00(4.95,7.06)	1.99(1.63,2.36)	-0.15(-0.21,-0.08)
Philippines	4.43(3.57,5.36)	1.54(1.23,1.91)	4.22(3.37,5.11)	3.67(2.91,4.58)	-0.17(-0.23,-0.11)
Poland	5.18(4.19,6.19)	2.25(1.80,2.69)	3.69(2.95,4.51)	2.29(1.79,2.81)	-1.07(-1.53,-0.61)
Portugal	3.92(3.09,4.84)	0.53(0.41,0.65)	3.52(2.75,4.39)	0.68(0.53,0.84)	-0.45(-0.59,-0.32)
Puerto Rico	3.41(2.64,4.25)	0.12(0.09,0.15)	3.13(2.41,3.91)	0.19(0.15,0.24)	-0.65(-1.05,-0.24)
Qatar	3.73(2.93,4.60)	0.01(0.01,0.01)	3.55(2.83,4.40)	0.07(0.06,0.10)	-0.25(-0.28,-0.22)
South Korea	2.42(1.86,2.97)	0.76(0.59,0.94)	2.03(1.59,2.52)	1.76(1.36,2.24)	-0.72(-1.85,0.43)
Moldova	3.63(2.84,4.50)	0.17(0.13,0.21)	3.66(2.87,4.51)	0.20(0.16,0.26)	-0.02(-0.10,0.07)
Romania	4.12(3.25,5.02)	1.17(0.91,1.45)	3.64(2.84,4.52)	1.19(0.92,1.48)	-0.79(-1.12,-0.47)
Russia	4.52(3.65,5.44)	8.22(6.43,10.03)	4.46(3.58,5.37)	9.89(7.67,12.02)	-0.08(-0.14,-0.02)
Rwanda	1.18(0.91,1.50)	0.04(0.03,0.05)	1.12(0.86,1.43)	0.08(0.06,0.11)	-0.23(-0.29,-0.17)
Saint Kitts and Nevis	3.62(2.80,4.52)	0.00(0.00,0.00)	3.23(2.49,4.02)	0.00(0.00,0.00)	-0.39(-0.42,-0.37)
Saint Lucia	3.75(2.91,4.64)	0.00(0.00,0.00)	3.45(2.67,4.29)	0.01(0.01,0.01)	-0.29(-0.31,-0.28)
Saint Vincent and the Grenadines	3.80(2.94,4.69)	0.00(0.00,0.00)	3.42(2.64,4.26)	0.00(0.00,0.01)	-0.40(-0.43,-0.37)
Samoa	4.33(3.44,5.24)	0.00(0.00,0.01)	4.18(3.32,5.06)	0.01(0.01,0.01)	-0.14(-0.15,-0.13)
San Marino	3.67(2.85,4.54)	0.00(0.00,0.00)	3.36(2.60,4.19)	0.00(0.00,0.00)	-0.34(-0.40,-0.28)
Sao Tome and Principe	1.80(1.40,2.29)	0.00(0.00,0.00)	1.69(1.31,2.14)	0.00(0.00,0.00)	-0.29(-0.35,-0.23)
Saudi Arabia	3.88(3.05,4.80)	0.31(0.24,0.39)	3.71(2.92,4.60)	1.03(0.79,1.30)	-0.17(-0.19,-0.16)
Senegal	2.42(1.89,3.08)	0.09(0.07,0.12)	2.29(1.79,2.88)	0.21(0.16,0.26)	-0.27(-0.39,-0.16)
Serbia	4.54(3.60,5.52)	0.54(0.42,0.67)	4.23(3.33,5.17)	0.60(0.46,0.74)	-0.33(-0.38,-0.28)
Seychelles	2.80(2.19,3.52)	0.00(0.00,0.00)	2.52(1.96,3.17)	0.00(0.00,0.00)	-0.32(-0.36,-0.29)
Sierra Leone	1.90(1.48,2.40)	0.04(0.03,0.05)	1.82(1.41,2.32)	0.08(0.06,0.10)	-0.21(-0.29,-0.13)
Singapore	2.27(1.76,2.80)	0.05(0.04,0.07)	2.02(1.56,2.54)	0.16(0.12,0.20)	-0.37(-0.38,-0.36)
Slovakia	4.37(3.47,5.36)	0.26(0.20,0.31)	3.81(2.99,4.76)	0.33(0.25,0.41)	-0.50(-0.68,-0.33)
Slovenia	4.95(3.99,5.98)	0.12(0.10,0.15)	4.47(3.56,5.42)	0.16(0.13,0.19)	-0.81(-1.22,-0.40)
Solomon Islands	4.47(3.55,5.39)	0.01(0.01,0.01)	4.33(3.41,5.24)	0.01(0.01,0.02)	-0.07(-0.09,-0.06)
Somalia	1.30(1.01,1.65)	0.04(0.03,0.06)	1.34(1.04,1.68)	0.12(0.10,0.16)	0.09(0.06,0.12)
South Africa	3.48(2.78,4.24)	1.03(0.81,1.28)	2.98(2.35,3.63)	1.54(1.20,1.92)	-0.64(-0.95,-0.33)
South Sudan	0.96(0.73,1.24)	0.03(0.02,0.04)	0.97(0.75,1.24)	0.05(0.04,0.06)	0.01(-0.01,0.02)
Spain	3.90(3.28,4.40)	2.07(1.74,2.36)	2.64(2.04,3.33)	2.12(1.63,2.65)	-1.66(-2.51,-0.80)
Sri Lanka	2.80(2.18,3.50)	0.33(0.25,0.42)	2.42(1.87,3.07)	0.63(0.48,0.81)	-1.43(-2.45,-0.40)
Sudan	4.60(3.67,5.61)	0.51(0.41,0.62)	4.31(3.42,5.30)	1.02(0.81,1.26)	-0.28(-0.31,-0.26)
Suriname	3.69(2.88,4.62)	0.01(0.01,0.01)	3.46(2.69,4.30)	0.02(0.02,0.03)	-0.29(-0.32,-0.27)
Sweden	3.12(2.49,3.87)	0.41(0.32,0.50)	3.25(2.53,4.07)	0.56(0.43,0.70)	-0.49(-1.37,0.39)
Switzerland	3.24(2.92,3.61)	0.30(0.27,0.34)	2.72(2.10,3.45)	0.41(0.31,0.51)	-0.94(-1.28,-0.60)
Syria	4.57(3.65,5.55)	0.29(0.23,0.37)	4.37(3.48,5.34)	0.60(0.47,0.77)	-0.24(-0.28,-0.20)
Taiwan (Province of China)	2.11(1.63,2.66)	0.34(0.25,0.44)	2.14(1.66,2.70)	0.84(0.64,1.06)	0.11(0.08,0.13)
Tajikistan	5.39(4.42,6.41)	0.17(0.14,0.21)	5.29(4.27,6.33)	0.35(0.28,0.44)	-0.17(-0.29,-0.04)
Thailand	3.04(2.38,3.80)	1.19(0.93,1.52)	2.64(2.05,3.35)	2.71(2.07,3.50)	-0.48(-0.50,-0.46)
Timor-Leste	3.18(2.47,3.93)	0.01(0.01,0.01)	2.83(2.22,3.56)	0.02(0.02,0.03)	-0.46(-0.51,-0.41)
Togo	1.87(1.45,2.35)	0.03(0.02,0.04)	1.81(1.40,2.30)	0.08(0.06,0.10)	-0.16(-0.21,-0.10)
Tokelau	4.26(3.37,5.18)	0.00(0.00,0.00)	4.01(3.14,4.94)	0.00(0.00,0.00)	-0.23(-0.24,-0.21)
Tonga	4.31(3.40,5.23)	0.00(0.00,0.00)	4.09(3.23,4.95)	0.00(0.00,0.00)	-0.16(-0.17,-0.15)

Trinidad and Tobago	3.64(2.82,4.56)	0.03(0.02,0.04)	3.24(2.50,4.09)	0.06(0.05,0.08)	-0.51(-0.55,-0.47)
Tunisia	4.39(3.52,5.36)	0.25(0.20,0.31)	4.04(3.20,4.96)	0.53(0.42,0.66)	-0.34(-0.57,-0.12)
Turkey	5.25(4.21,6.29)	2.23(1.79,2.73)	4.78(3.82,5.80)	4.43(3.52,5.44)	-0.19(-0.43,0.05)
Turkmenistan	5.16(4.14,6.15)	0.12(0.09,0.14)	4.79(3.84,5.77)	0.21(0.17,0.27)	-0.37(-0.50,-0.24)
Tuvalu	4.38(3.46,5.28)	0.00(0.00,0.00)	4.14(3.22,5.08)	0.00(0.00,0.00)	-0.16(-0.17,-0.15)
Uganda	1.18(0.91,1.51)	0.10(0.07,0.12)	1.10(0.84,1.40)	0.21(0.16,0.27)	-0.28(-0.29,-0.26)
Ukraine	4.33(3.46,5.25)	3.02(2.37,3.68)	4.32(3.46,5.23)	3.04(2.37,3.68)	0.02(-0.07,0.11)
United Arab Emirates	3.72(2.91,4.59)	0.03(0.03,0.04)	3.79(2.99,4.67)	0.31(0.22,0.40)	-0.01(-0.45,0.44)
UK	4.07(3.21,4.99)	3.29(2.59,3.98)	3.67(2.87,4.53)	3.92(3.06,4.83)	-0.63(-1.12,-0.14)
Tanzania	1.15(0.88,1.45)	0.15(0.12,0.19)	1.09(0.83,1.38)	0.33(0.25,0.43)	-0.22(-0.25,-0.18)
USA	3.86(2.94,4.89)	11.27(8.67,14.10)	3.03(2.36,3.81)	14.29(10.96,18.16)	-1.26(-1.67,-0.84)
Virgin Islands US	2.80(2.15,3.55)	0.00(0.00,0.00)	2.51(1.96,3.21)	0.00(0.00,0.01)	-0.92(-1.70,-0.13)
Uruguay	3.94(3.11,4.81)	0.15(0.12,0.18)	3.44(2.67,4.30)	0.16(0.13,0.20)	-0.43(-0.58,-0.28)
Uzbekistan	5.27(4.31,6.28)	0.68(0.55,0.83)	5.03(4.04,6.02)	1.35(1.05,1.72)	-0.25(-0.35,-0.14)
Vanuatu	4.35(3.46,5.25)	0.00(0.00,0.00)	4.21(3.33,5.10)	0.01(0.01,0.01)	-0.11(-0.12,-0.10)
Venezuela	3.71(2.92,4.65)	0.42(0.33,0.53)	3.52(2.77,4.39)	1.08(0.84,1.35)	-0.26(-0.28,-0.23)
Vietnam	2.75(2.13,3.44)	1.17(0.90,1.47)	2.32(1.81,2.92)	2.25(1.73,2.90)	-0.50(-1.09,0.11)
Yemen	4.61(3.72,5.59)	0.29(0.23,0.35)	4.53(3.61,5.54)	0.79(0.63,0.98)	-0.16(-0.20,-0.11)
Zambia	1.15(0.88,1.47)	0.04(0.03,0.05)	1.08(0.82,1.37)	0.1(0.07,0.13)	-0.31(-0.66,0.05)
Zimbabwe	2.44(1.92,3.03)	0.15(0.12,0.19)	2.43(1.90,3.00)	0.25(0.19,0.31)	0.03(-0.21,0.27)

ASIR, age-standardized incidence rate; No., number; UI, uncertainty interval; EAPC, estimated annual percentage change; CI, confidential interval.

Supplementary table 2. Prevalence and age-standardized prevalence rate per 1000 people for edentulism in 1990 and 2019, and its estimated annual percentage change in 204 countries and territories from 1990 to 2019.

	1990		2019		EAPC (95%CI)
	ASPR/1000 (95%UI)	Prevalence*10^5 (95%UI)	ASPR/1000 (95%UI)	Prevalence*10^5 (95%UI)	From 1990 to 2019
Afghanistan	74.37(59.52,94.51)	5.28(4.19,6.80)	73.43(58.38,93.12)	10.67(8.35,13.70)	-0.15(-0.24,-0.05)
Albania	72.11(57.43,91.89)	1.54(1.22,1.97)	63.63(50.17,81.04)	2.66(2.08,3.42)	-0.56(-0.62,-0.51)
Algeria	68.33(54.54,86.79)	8.86(7.03,11.32)	65.22(51.78,83.08)	23.10(18.25,29.69)	-0.24(-0.27,-0.21)
American Samoa	50.61(39.83,64.24)	0.01(0.01,0.01)	50.56(39.40,64.16)	0.02(0.02,0.03)	-0.01(-0.03,0.00)
Andorra	50.10(39.50,64.35)	0.03(0.02,0.04)	47.22(37.10,60.93)	0.06(0.05,0.08)	-0.24(-0.27,-0.20)
Angola	37.27(29.33,48.08)	2.01(1.52,2.65)	33.38(26.25,43.00)	5.25(3.97,6.96)	-0.55(-0.63,-0.47)
Antigua and Barbuda	50.17(39.17,64.42)	0.03(0.02,0.03)	45.16(34.79,58.29)	0.05(0.03,0.06)	-0.37(-0.39,-0.35)
Argentina	52.60(41.14,66.79)	16.93(13.17,21.61)	45.53(35.43,58.69)	24.49(19.04,31.47)	-0.48(-0.52,-0.44)
Armenia	88.58(71.10,110.90)	2.43(1.93,3.08)	82.12(65.49,103.33)	3.35(2.67,4.24)	-0.45(-0.59,-0.31)
Australia	103.67(100.46,106.28)	19.89(19.29,20.39)	64.03(50.75,81.81)	25.05(19.71,31.79)	-0.68(-1.35,0.00)
Austria	67.24(53.64,85.78)	7.72(6.10,9.77)	59.69(47.20,75.83)	9.90(7.78,12.52)	-0.72(-1.14,-0.30)
Azerbaijan	83.90(68.08,104.88)	4.27(3.43,5.43)	78.14(62.71,98.77)	7.46(5.87,9.61)	-0.46(-0.65,-0.28)
Bahamas	46.74(36.28,60.49)	0.07(0.06,0.09)	44.20(34.21,57.15)	0.17(0.13,0.22)	-0.23(-0.25,-0.20)
Bahrain	60.85(47.72,76.90)	0.14(0.11,0.18)	57.94(45.74,74.54)	0.74(0.57,0.98)	-0.21(-0.23,-0.18)
Bangladesh	11.74(9.04,15.12)	5.20(3.95,6.75)	9.95(7.48,12.66)	12.41(9.25,16.08)	-0.58(-0.60,-0.56)
Barbados	50.26(38.87,64.38)	0.15(0.11,0.18)	46.85(36.61,60.82)	0.23(0.18,0.30)	-0.28(-0.31,-0.26)
Belarus	57.18(45.24,73.99)	7.25(5.74,9.46)	53.36(42.28,67.86)	8.14(6.42,10.39)	-0.33(-0.39,-0.27)
Belgium	68.32(54.08,87.23)	10.20(8.06,12.92)	60.61(47.82,76.78)	12.74(10.05,16.04)	-0.53(-0.78,-0.27)
Belize	53.75(41.65,68.76)	0.05(0.04,0.06)	48.88(38.25,63.55)	0.13(0.10,0.17)	-0.34(-0.37,-0.31)
Benin	29.52(23.11,37.50)	0.66(0.51,0.85)	27.63(21.60,35.54)	1.59(1.22,2.08)	-0.30(-0.34,-0.26)
Bermuda	46.55(36.19,60.46)	0.03(0.02,0.04)	42.55(32.94,54.99)	0.05(0.04,0.07)	-0.37(-0.40,-0.34)
Bhutan	18.63(14.43,23.55)	0.04(0.03,0.06)	15.52(11.95,19.78)	0.08(0.06,0.11)	-0.65(-0.66,-0.64)
Bolivia	105.41(85.72,128.71)	3.54(2.87,4.39)	96.08(77.42,120.52)	8.74(7.01,11.01)	-0.33(-0.35,-0.31)
Bosnia and Herzegovina	78.85(63.15,99.92)	3.23(2.57,4.17)	67.78(53.47,85.98)	3.90(3.03,4.98)	-1.05(-1.62,-0.48)
Botswana	61.82(48.75,77.97)	0.47(0.36,0.61)	55.42(43.52,70.10)	1.06(0.80,1.38)	-0.38(-0.38,-0.37)
Brazil	95.18(77.43,116.12)	85.71(69.28,106.08)	91.94(74.43,113.32)	218.80(176.14,270.89)	-0.10(-0.48,0.29)
Brunei	27.69(21.57,34.50)	0.03(0.02,0.04)	27.41(21.20,33.98)	0.08(0.06,0.11)	-0.09(-0.12,-0.06)
Bulgaria	62.02(49.04,79.43)	7.54(5.88,9.78)	54.92(43.22,71.25)	7.62(5.88,9.92)	-0.73(-0.99,-0.47)
Burkina Faso	19.90(15.46,25.57)	0.91(0.70,1.19)	18.26(14.18,23.37)	1.87(1.43,2.45)	-0.22(-0.50,0.06)
Burundi	18.88(14.79,24.18)	0.51(0.39,0.66)	19.19(14.75,25.05)	1.07(0.79,1.44)	0.05(0.01,0.08)
Cape Verde	28.71(22.60,36.71)	0.07(0.05,0.08)	25.20(19.79,32.37)	0.12(0.09,0.15)	-0.56(-0.60,-0.52)
Cambodia	49.32(38.80,63.18)	2.23(1.74,2.89)	42.47(33.47,54.30)	5.04(3.94,6.50)	-0.53(-0.59,-0.47)
Cameroon	27.31(21.31,34.87)	1.35(1.05,1.77)	26.39(20.78,33.70)	3.73(2.83,4.87)	-0.20(-0.26,-0.14)
Canada	43.86(34.64,56.03)	14.04(11.11,17.88)	35.34(27.56,45.55)	21.77(16.98,27.76)	-0.53(-0.81,-0.24)
Central African Republic	40.30(31.58,51.34)	0.61(0.47,0.81)	40.83(31.57,51.73)	1.24(0.93,1.61)	-0.01(-0.03,0.01)
Chad	39.46(31.15,50.49)	1.21(0.95,1.56)	36.02(28.54,46.04)	2.42(1.88,3.18)	-0.39(-0.65,-0.13)
Chile	47.93(36.97,62.03)	4.73(3.64,6.12)	40.21(31.21,51.71)	9.68(7.49,12.46)	-0.57(-0.64,-0.51)
China	35.20(27.37,44.63)	275.09(212.43,357.72)	33.27(25.89,42.27)	641.88(495.73,829.32)	0.22(-0.45,0.90)
Colombia	48.07(37.96,61.56)	8.86(6.92,11.38)	43.19(33.83,55.19)	22.82(17.89,29.14)	-0.41(-0.44,-0.38)
Comoros	22.43(17.60,28.83)	0.05(0.04,0.07)	22.42(17.45,28.84)	0.12(0.09,0.16)	-0.01(-0.12,0.11)
Congo	35.10(27.4,44.79)	0.47(0.36,0.61)	33.89(26.49,43.60)	1.20(0.91,1.57)	-0.20(-0.27,-0.13)
Cook Islands	52.63(41.34,66.86)	0.01(0.01,0.01)	48.75(37.96,62.60)	0.01(0.01,0.02)	-0.24(-0.25,-0.22)
Costa Rica	58.59(46.69,74.67)	1.07(0.85,1.37)	53.58(42.04,68.61)	2.77(2.18,3.55)	-0.34(-0.35,-0.33)

Croatia	65.14(51.13,83.32)	4.11(3.18,5.30)	57.74(45.63,74.19)	4.77(3.73,6.16)	-1.08(-1.65,-0.50)
Cuba	56.86(44.34,73.70)	5.84(4.55,7.54)	52.28(46.42,59.08)	9.76(8.69,11.00)	-0.37(-0.44,-0.30)
Cyprus	56.34(44.17,71.60)	0.47(0.37,0.60)	51.85(40.56,66.33)	0.99(0.77,1.27)	-0.31(-0.33,-0.28)
Czech	66.84(52.78,85.44)	9.03(7.09,11.58)	58.47(45.71,75.44)	11.64(9.03,15.01)	-0.60(-0.88,-0.32)
Cote d'Ivoire	38.51(30.55,48.96)	1.97(1.50,2.61)	37.34(29.27,47.65)	4.97(3.81,6.53)	-0.13(-0.25,-0.01)
North Korea	29.56(22.90,37.10)	4.15(3.23,5.34)	33.11(25.93,41.38)	10.19(7.96,12.85)	0.40(0.36,0.43)
DR Congo	38.94(30.44,49.65)	7.90(6.00,10.28)	39.76(30.80,50.73)	19.77(14.96,26.06)	0.05(-0.07,0.16)
Denmark	51.75(40.31,67.01)	4.07(3.16,5.24)	44.04(34.19,56.41)	4.78(3.70,6.09)	-1.21(-2.13,-0.27)
Djibouti	16.53(12.83,21.26)	0.03(0.02,0.04)	16.59(12.75,21.41)	0.12(0.09,0.17)	-0.01(-0.06,0.04)
Dominica	53.94(41.93,68.63)	0.04(0.03,0.05)	47.67(37.10,61.22)	0.04(0.03,0.05)	-0.47(-0.50,-0.45)
Dominican Republic	65.28(51.27,83.05)	2.44(1.93,3.13)	58.31(45.74,74.18)	5.39(4.23,6.90)	-0.40(-0.53,-0.28)
Ecuador	92.78(74.85,115.48)	5.13(4.16,6.45)	85.08(68.07,106.12)	13.03(10.43,16.27)	-0.49(-0.81,-0.18)
Egypt	71.04(56.05,89.30)	22.07(17.41,28.19)	65.79(52.42,84.07)	44.55(35.27,57.78)	-0.30(-0.31,-0.28)
El Salvador	61.40(48.81,77.62)	1.87(1.48,2.38)	56.91(44.83,72.68)	3.37(2.66,4.29)	-0.27(-0.27,-0.26)
Equatorial Guinea	39.35(30.73,50.44)	0.09(0.07,0.12)	27.93(21.84,35.94)	0.21(0.16,0.28)	-1.55(-1.75,-1.35)
Eritrea	19.03(14.78,24.74)	0.23(0.17,0.32)	17.89(13.79,23.34)	0.60(0.44,0.81)	-0.15(-0.21,-0.09)
Estonia	56.67(45.24,72.79)	1.13(0.90,1.45)	51.87(40.66,66.37)	1.27(0.99,1.62)	-0.77(-1.15,-0.38)
Eswatini	56.47(44.50,72.47)	0.24(0.18,0.31)	51.84(40.89,66.30)	0.41(0.32,0.55)	-0.28(-0.31,-0.26)
Ethiopia	19.48(15.08,25.22)	4.35(3.26,5.84)	18.10(13.92,23.37)	8.88(6.67,11.83)	-0.29(-0.53,-0.06)
Fiji	57.26(45.60,73.16)	0.20(0.16,0.26)	53.45(41.88,68.42)	0.38(0.29,0.49)	-0.22(-0.23,-0.21)
Finland	73.93(61.77,90.06)	5.23(4.38,6.33)	53.58(42.06,69.14)	6.07(4.74,7.77)	-1.60(-2.23,-0.98)
France	56.49(44.38,71.93)	45.68(35.80,58.03)	50.38(39.55,64.73)	63.32(49.33,80.45)	-0.34(-1.14,0.47)
Gabon	31.00(24.19,39.89)	0.19(0.15,0.25)	30.08(23.54,38.50)	0.39(0.30,0.50)	-0.11(-0.14,-0.09)
Gambia	29.24(22.96,37.43)	0.12(0.09,0.16)	28.43(22.47,36.32)	0.32(0.25,0.41)	-0.16(-0.20,-0.11)
Georgia	71.99(57.62,92.27)	4.36(3.46,5.66)	70.59(55.99,89.42)	4.09(3.23,5.17)	-0.27(-0.45,-0.09)
Germany	53.02(41.53,68.22)	65.55(50.86,84.18)	45.81(35.62,59.30)	81.05(62.57,104.25)	-1.01(-1.68,-0.33)
Ghana	26.32(20.59,33.87)	1.86(1.43,2.45)	23.80(18.34,30.43)	4.34(3.33,5.66)	-0.25(-0.41,-0.09)
Greece	57.77(45.55,73.08)	8.62(6.75,10.93)	52.89(41.57,67.43)	11.43(8.85,14.35)	-0.68(-1.20,-0.15)
Greenland	44.28(35.06,57.14)	0.02(0.01,0.02)	38.21(30.21,49.32)	0.03(0.02,0.03)	-0.53(-0.55,-0.51)
Grenada	53.82(41.82,69.09)	0.04(0.03,0.05)	47.23(36.64,61.41)	0.05(0.04,0.07)	-0.48(-0.49,-0.47)
Guam	47.28(36.85,60.13)	0.03(0.03,0.05)	45.96(35.57,58.58)	0.09(0.07,0.11)	-0.40(-0.69,-0.10)
Guatemala	70.38(56.52,89.00)	2.74(2.19,3.49)	66.64(53.35,84.33)	7.75(6.19,9.78)	-0.21(-0.22,-0.20)
Guinea	28.93(22.70,36.99)	1.01(0.79,1.32)	27.78(21.76,35.77)	1.74(1.34,2.28)	-0.17(-0.21,-0.12)
Guinea-Bissau	29.93(23.55,38.38)	0.14(0.11,0.18)	28.59(22.51,36.75)	0.25(0.19,0.33)	-0.16(-0.20,-0.13)
Guyana	55.70(43.46,71.04)	0.21(0.16,0.27)	49.91(38.94,64.26)	0.31(0.24,0.40)	-0.40(-0.41,-0.39)
Haiti	57.45(44.65,73.44)	1.83(1.42,2.38)	55.57(43.33,72.11)	3.86(3.03,5.02)	-0.14(-0.15,-0.13)
Honduras	62.75(49.97,80.49)	1.36(1.08,1.74)	59.09(46.77,76.45)	3.76(2.99,4.83)	-0.24(-0.25,-0.23)
Hungary	68.41(53.77,87.20)	9.86(7.67,12.62)	60.06(47.36,77.59)	11.02(8.60,14.12)	-0.35(-0.67,-0.04)
Iceland	55.70(43.99,70.91)	0.16(0.12,0.20)	48.90(37.52,62.71)	0.26(0.20,0.33)	-0.51(-0.54,-0.48)
India	34.69(26.32,44.69)	149.88(109.85,202.85)	31.42(24.01,40.08)	349.06(262.03,452.25)	1.91(0.97,2.86)
Indonesia	50.55(40.06,64.40)	49.32(38.74,64.00)	45.29(35.81,57.78)	96.50(75.42,125.74)	-0.33(-0.37,-0.28)
Iran	55.43(44.02,70.80)	15.18(11.96,19.78)	52.57(41.57,67.53)	40.17(31.85,51.62)	-0.77(-1.34,-0.20)
Iraq	68.02(54.09,87.36)	5.62(4.47,7.27)	64.57(50.97,82.79)	16.29(12.90,21.05)	-0.32(-0.38,-0.26)
Ireland	77.63(63.78,94.36)	3.12(2.54,3.79)	63.74(50.08,81.26)	4.58(3.60,5.84)	-0.60(-0.94,-0.25)
Israel	65.92(51.81,83.45)	3.19(2.51,4.03)	58.32(46.26,74.02)	6.54(5.14,8.29)	-0.42(-0.46,-0.37)
Italy	51.01(40.10,65.07)	44.34(34.68,56.62)	48.64(38.07,62.48)	64.01(49.97,80.69)	-0.62(-1.12,-0.11)
Jamaica	53.46(41.36,68.15)	0.95(0.74,1.21)	49.91(38.53,63.81)	1.49(1.15,1.91)	-0.22(-0.23,-0.20)
Japan	45.57(35.87,57.10)	75.76(59.56,95.27)	37.71(29.84,47.34)	123.77(96.29,152.37)	-0.54(-1.34,0.27)
Jordan	70.22(56.10,88.46)	1.05(0.83,1.35)	66.49(52.75,84.49)	4.88(3.83,6.33)	-0.29(-0.33,-0.25)
Kazakhstan	93.03(75.34,115.15)	11.92(9.54,14.96)	86.49(69.74,108.34)	15.07(12.04,19.15)	-0.31(-0.45,-0.17)

Kenya	16.22(12.69,20.89)	1.52(1.16,2.03)	16.17(12.65,20.92)	4.16(3.15,5.62)	-0.40(-0.60,-0.19)
Kiribati	61.01(48.53,77.42)	0.02(0.02,0.03)	61.69(48.94,78.45)	0.04(0.03,0.05)	0.06(0.04,0.08)
Kuwait	60.47(47.63,76.81)	0.48(0.37,0.63)	57.79(45.87,73.84)	1.90(1.47,2.46)	-0.20(-0.23,-0.17)
Kyrgyzstan	85.81(69.19,106.73)	2.65(2.13,3.32)	85.05(68.71,106.46)	4.02(3.20,5.09)	-0.13(-0.26,0.00)
Laos	30.47(23.58,39.46)	0.61(0.47,0.8)	25.19(19.43,32.33)	1.08(0.83,1.40)	-0.69(-1.32,-0.06)
Latvia	45.08(35.20,57.66)	1.57(1.23,2.03)	42.79(33.31,55.18)	1.60(1.24,2.05)	-0.78(-1.32,-0.24)
Lebanon	69.84(55.70,88.55)	1.62(1.30,2.07)	65.43(51.58,83.66)	3.44(2.70,4.39)	-0.28(-0.30,-0.26)
Lesotho	71.61(56.32,91.18)	0.85(0.65,1.10)	65.34(51.62,82.69)	1.08(0.83,1.40)	-0.31(-0.33,-0.29)
Liberia	29.86(23.41,38.27)	0.34(0.27,0.45)	29.72(23.36,38.21)	0.74(0.56,0.97)	-0.24(-0.36,-0.12)
Libya	62.21(49.26,78.98)	1.24(0.98,1.60)	64.18(51.01,81.80)	3.58(2.82,4.58)	0.00(-0.06,0.06)
Lithuania	46.07(36.15,59.08)	2.04(1.61,2.63)	42.10(32.86,54.42)	2.27(1.77,2.92)	-0.81(-1.21,-0.40)
Luxembourg	65.11(51.69,82.37)	0.35(0.28,0.44)	56.07(43.77,72.12)	0.54(0.42,0.69)	-0.87(-1.26,-0.48)
Madagascar	17.86(13.72,23.20)	1.04(0.79,1.38)	18.01(13.90,23.32)	2.41(1.79,3.24)	0.02(-0.01,0.04)
Malawi	22.33(17.47,28.59)	1.01(0.77,1.34)	21.20(16.41,27.49)	1.88(1.41,2.51)	-0.21(-0.22,-0.19)
Malaysia	62.24(49.45,78.97)	5.99(4.73,7.66)	53.98(42.47,68.90)	14.6(11.42,18.75)	-0.84(-1.55,-0.12)
Maldives	40.58(31.52,52.38)	0.04(0.03,0.05)	35.36(27.54,45.02)	0.11(0.09,0.15)	-0.48(-0.51,-0.44)
Mali	23.43(18.20,30.33)	1.02(0.80,1.35)	21.38(16.68,27.44)	2.07(1.60,2.71)	-0.39(-0.68,-0.09)
Malta	49.32(38.46,63.81)	0.21(0.16,0.27)	41.49(32.20,53.67)	0.37(0.28,0.48)	-0.70(-0.82,-0.59)
Marshall Islands	58.86(46.45,74.31)	0.01(0.01,0.01)	56.53(44.69,71.49)	0.02(0.01,0.02)	-0.11(-0.13,-0.10)
Mauritania	37.86(29.89,48.26)	0.42(0.33,0.54)	35.68(28.04,45.87)	0.83(0.65,1.09)	-0.26(-0.47,-0.06)
Mauritius	65.13(52.04,82.66)	0.49(0.39,0.63)	57.12(45.50,73.01)	0.98(0.77,1.27)	-0.39(-0.97,0.20)
Mexico	68.02(54.16,86.79)	30.67(24.50,39.26)	65.06(51.68,83.16)	77.48(61.54,99.53)	-0.55(-0.81,-0.28)
Micronesia	59.58(47.25,75.85)	0.03(0.02,0.03)	57.15(45.23,72.69)	0.04(0.03,0.05)	-0.16(-0.16,-0.15)
Monaco	47.32(36.85,61.30)	0.03(0.02,0.04)	41.74(32.59,54.21)	0.04(0.03,0.05)	-0.42(-0.43,-0.41)
Mongolia	85.67(69.16,106.92)	0.94(0.75,1.18)	78.37(63.18,98.45)	1.90(1.49,2.42)	-0.42(-0.53,-0.30)
Montenegro	67.81(53.13,86.76)	0.42(0.33,0.54)	63.85(50.50,81.46)	0.61(0.48,0.79)	-0.33(-0.40,-0.26)
Morocco	81.86(65.84,104.00)	11.92(9.51,15.30)	74.63(59.45,93.71)	24.21(19.05,30.66)	-0.35(-0.41,-0.30)
Mozambique	20.56(16.03,26.42)	1.39(1.06,1.84)	18.71(14.36,24.16)	2.47(1.85,3.29)	-0.40(-0.42,-0.38)
Myanmar	30.47(23.56,39.59)	6.84(5.27,9.11)	24.36(18.90,31.09)	10.93(8.39,14.10)	-0.92(-1.81,-0.03)
Namibia	87.76(68.76,112.45)	0.80(0.62,1.03)	80.07(62.77,102.10)	1.48(1.14,1.92)	-0.32(-0.74,0.11)
Nauru	51.81(41.04,65.86)	0.00(0.00,0.00)	52.92(41.75,67.82)	0.00(0.00,0.00)	0.11(-0.02,0.24)
Nepal	14.53(11.19,18.56)	1.24(0.94,1.65)	12.02(9.17,15.51)	2.50(1.88,3.28)	-0.64(-0.86,-0.42)
Netherlands	81.14(64.97,101.28)	15.84(12.73,19.64)	71.35(56.45,89.60)	22.55(17.80,28.34)	-0.76(-1.34,-0.18)
New Zealand	98.35(76.29,127.88)	3.77(2.93,4.87)	71.79(55.55,93.05)	5.23(4.05,6.74)	-0.89(-1.26,-0.52)
Nicaragua	62.55(49.66,79.73)	1.02(0.81,1.32)	59.26(47.34,74.99)	2.76(2.19,3.49)	-0.24(-0.26,-0.21)
Niger	29.94(23.73,38.25)	0.97(0.75,1.28)	29.22(23.06,37.28)	2.66(2.06,3.46)	-0.14(-0.19,-0.08)
Nigeria	18.83(14.57,24.03)	8.60(6.62,11.23)	17.12(13.27,21.97)	16.41(12.48,21.77)	-0.66(-1.56,0.24)
Niue	54.82(43.24,69.83)	0.00(0.00,0.00)	51.91(40.65,66.08)	0.00(0.00,0.00)	-0.21(-0.24,-0.19)
North Macedonia	72.76(57.79,92.49)	1.36(1.06,1.74)	66.75(53.04,85.72)	2.10(1.64,2.73)	-0.39(-0.44,-0.33)
Northern Mariana Islands	47.56(36.97,60.66)	0.01(0.01,0.01)	48.22(37.53,61.69)	0.02(0.02,0.03)	0.08(0.05,0.11)
Norway	52.86(41.35,67.39)	3.47(2.71,4.39)	49.24(38.58,63.10)	4.48(3.49,5.73)	-0.24(-1.76,1.30)
Oman	61.27(48.47,77.89)	0.49(0.38,0.64)	57.79(45.57,73.00)	1.42(1.10,1.86)	-0.25(-0.28,-0.23)
Pakistan	29.55(23.35,37.14)	16.50(12.83,20.85)	28.45(22.52,35.54)	30.87(24.01,39.23)	-0.08(-0.86,0.71)
Palau	53.42(41.72,68.36)	0.01(0.00,0.01)	51.06(40.04,64.98)	0.01(0.01,0.01)	-0.15(-0.16,-0.13)
Palestine	74.79(59.44,94.16)	0.69(0.55,0.87)	68.98(54.41,87.55)	1.83(1.43,2.33)	-0.29(-0.31,-0.26)
Panama	58.10(46.23,74.02)	0.90(0.71,1.15)	51.54(40.65,67.05)	2.14(1.69,2.78)	-0.42(-0.44,-0.40)
Papua New Guinea	59.88(47.54,75.49)	1.06(0.83,1.36)	57.38(45.42,72.90)	2.58(2.02,3.36)	-0.12(-0.15,-0.10)
Paraguay	71.79(56.58,89.73)	1.59(1.26,1.99)	64.02(50.19,80.47)	3.55(2.78,4.49)	-0.11(-0.72,0.50)
Peru	108.21(89.00,130.98)	13.51(10.98,16.44)	99.73(81.09,123.84)	32.20(26.20,40.13)	-0.19(-0.27,-0.10)

Philippines	65.78(52.82,82.80)	20.22(16.08,25.79)	62.90(50.35,79.40)	49.28(39.07,63.04)	-0.14(-0.18,-0.11)
Poland	81.15(64.95,102.62)	35.16(27.93,44.72)	55.32(43.71,70.31)	36.49(28.80,46.36)	-1.29(-1.88,-0.70)
Portugal	58.00(45.84,73.71)	7.98(6.25,10.17)	51.32(40.41,65.98)	11.40(8.87,14.52)	-0.49(-0.60,-0.38)
Puerto Rico	46.90(36.26,60.62)	1.71(1.32,2.21)	42.93(33.19,55.69)	2.97(2.27,3.83)	-0.64(-1.00,-0.27)
Qatar	55.99(44.00,71.57)	0.10(0.08,0.14)	53.45(42.12,68.38)	0.85(0.63,1.15)	-0.27(-0.30,-0.23)
South Korea	30.94(24.14,38.10)	8.98(6.97,11.32)	25.81(20.01,32.18)	22.25(17.17,27.89)	-0.88(-2.03,0.29)
Moldova	54.79(43.18,69.65)	2.38(1.86,3.07)	55.53(44.09,70.54)	3.11(2.46,3.97)	0.00(-0.11,0.10)
Romania	60.68(48.14,77.58)	16.69(13.08,21.57)	52.81(41.41,68.56)	18.81(14.64,24.53)	-0.84(-1.15,-0.52)
Russia	71.21(57.56,89.87)	125.38(101.03,159.15)	69.80(56.27,88.05)	158.49(127.08,201.20)	-0.11(-0.17,-0.05)
Rwanda	19.14(14.85,24.75)	0.64(0.49,0.85)	18.11(13.99,23.41)	1.29(0.97,1.71)	-0.25(-0.31,-0.18)
Saint Kitts and Nevis	50.89(39.59,65.45)	0.02(0.01,0.02)	44.67(34.36,57.76)	0.03(0.02,0.04)	-0.45(-0.48,-0.43)
Saint Lucia	52.85(41.12,67.90)	0.05(0.04,0.06)	48.01(37.26,61.40)	0.10(0.08,0.13)	-0.34(-0.36,-0.32)
Saint Vincent and the Grenadines	53.97(42.31,69.15)	0.04(0.03,0.05)	47.28(36.49,61.34)	0.06(0.05,0.08)	-0.50(-0.53,-0.47)
Samoa	59.54(47.18,75.47)	0.05(0.04,0.07)	57.15(45.34,72.62)	0.08(0.06,0.10)	-0.18(-0.20,-0.16)
San Marino	53.41(42.24,67.90)	0.02(0.01,0.02)	48.60(37.84,62.63)	0.03(0.02,0.04)	-0.37(-0.44,-0.31)
Sao Tome and Principe	28.02(21.96,35.74)	0.02(0.01,0.02)	26.21(20.50,33.36)	0.03(0.02,0.04)	-0.29(-0.35,-0.23)
Saudi Arabia	59.31(46.74,74.98)	4.01(3.15,5.19)	56.92(44.77,73.33)	12.96(9.90,17.13)	-0.17(-0.19,-0.15)
Senegal	40.49(32.00,52.35)	1.48(1.15,1.93)	38.29(30.20,48.98)	3.28(2.56,4.30)	-0.24(-0.42,-0.05)
Serbia	68.44(54.23,87.49)	7.68(5.99,9.95)	63.61(50.24,80.57)	9.71(7.55,12.44)	-0.36(-0.41,-0.30)
Seychelles	38.39(29.95,49.36)	0.02(0.02,0.03)	34.30(26.76,43.92)	0.04(0.03,0.05)	-0.34(-0.38,-0.31)
Sierra Leone	29.99(23.69,38.08)	0.62(0.49,0.80)	28.86(22.63,36.93)	1.22(0.95,1.60)	-0.18(-0.27,-0.10)
Singapore	28.95(22.64,35.88)	0.65(0.51,0.83)	25.95(20.18,32.39)	1.99(1.55,2.51)	-0.36(-0.36,-0.35)
Slovakia	65.03(51.04,83.12)	3.85(3.01,4.94)	55.57(43.59,71.69)	4.98(3.87,6.46)	-0.56(-0.87,-0.24)
Slovenia	77.85(62.09,98.66)	1.89(1.49,2.39)	68.81(54.75,86.37)	2.77(2.19,3.47)	-0.90(-1.32,-0.48)
Solomon Islands	62.00(49.37,78.10)	0.08(0.06,0.10)	59.99(47.24,75.91)	0.17(0.14,0.22)	-0.08(-0.10,-0.05)
Somalia	21.42(16.55,27.79)	0.67(0.49,0.90)	22.44(17.50,29)	1.91(1.42,2.55)	0.15(0.12,0.19)
South Africa	77.68(60.49,99.39)	20.23(15.50,26.22)	59.70(46.66,76.23)	30.37(23.24,38.98)	-0.96(-1.40,-0.53)
South Sudan	14.69(11.34,18.91)	0.40(0.31,0.53)	15.02(11.61,19.59)	0.68(0.51,0.90)	0.05(0.03,0.06)
Spain	54.07(44.82,61.61)	29.81(24.59,33.99)	36.52(28.65,47.18)	32.76(25.49,41.95)	-1.99(-3.13,-0.85)
Sri Lanka	37.76(29.47,48.22)	4.08(3.18,5.27)	32.48(25.06,42.23)	8.14(6.24,10.71)	-1.48(-2.51,-0.43)
Sudan	74.13(59.15,94.09)	7.33(5.83,9.31)	68.66(54.32,87.65)	14.23(11.24,18.28)	-0.33(-0.36,-0.30)
Suriname	51.63(40.15,66.61)	0.13(0.10,0.17)	48.32(37.61,62.25)	0.29(0.22,0.37)	-0.29(-0.31,-0.26)
Sweden	44.09(34.70,56.11)	6.33(4.94,8.00)	45.57(35.05,58.86)	8.87(6.80,11.32)	-0.45(-1.20,0.29)
Switzerland	46.27(42.99,49.74)	4.68(4.33,5.08)	37.97(29.49,49.31)	6.22(4.81,8.03)	-1.17(-1.65,-0.69)
Syria	73.44(58.29,92.39)	4.13(3.27,5.29)	70.03(55.75,88.80)	8.92(7.01,11.55)	-0.26(-0.31,-0.21)
Taiwan (Province of China)	24.51(18.96,31.16)	3.61(2.78,4.70)	24.98(19.28,31.59)	9.80(7.55,12.45)	0.11(0.07,0.14)
Tajikistan	87.51(70.66,109.03)	2.55(2.06,3.19)	85.31(69.06,106.06)	4.64(3.64,5.92)	-0.22(-0.38,-0.06)
Thailand	42.20(32.77,54.45)	15.08(11.67,19.72)	35.97(28.05,46.79)	36.20(28.08,47.30)	-0.54(-0.56,-0.52)
Timor-Leste	44.14(34.73,55.78)	0.13(0.10,0.17)	38.86(30.25,49.85)	0.32(0.24,0.41)	-0.52(-0.57,-0.46)
Togo	29.29(22.91,37.06)	0.44(0.33,0.57)	28.65(22.36,36.81)	1.21(0.93,1.58)	-0.11(-0.15,-0.06)
Tokelau	58.79(46.72,74.11)	0.00(0.00,0.00)	54.14(42.27,68.86)	0.00(0.00,0.00)	-0.30(-0.32,-0.29)
Tonga	59.21(46.71,74.68)	0.03(0.03,0.04)	55.58(43.78,70.65)	0.04(0.03,0.06)	-0.20(-0.21,-0.19)
Trinidad and Tobago	50.9(39.42,65.89)	0.43(0.33,0.56)	44.66(34.60,57.83)	0.83(0.64,1.09)	-0.58(-0.63,-0.53)
Tunisia	70.13(56.08,88.66)	3.68(2.93,4.69)	63.73(50.53,81.34)	8.14(6.40,10.41)	-0.39(-0.58,-0.20)
Turkey	88.52(71.18,110.20)	33.74(26.9,42.29)	79.03(63.25,99.28)	71.13(56.83,89.68)	-0.31(-0.53,-0.09)
Turkmenistan	82.68(66.42,102.74)	1.64(1.30,2.08)	75.01(60.68,94.56)	2.96(2.36,3.78)	-0.48(-0.64,-0.32)
Tuvalu	60.86(47.96,76.64)	0.00(0.00,0.01)	56.77(44.84,72.46)	0.01(0.00,0.01)	-0.20(-0.21,-0.18)
Uganda	18.96(14.76,24.51)	1.43(1.08,1.89)	17.62(13.68,22.90)	3.08(2.30,4.15)	-0.29(-0.30,-0.27)

Ukraine	67.54(54.07,85.70)	46.74(37.46,59.94)	67.21(53.84,84.72)	49.28(39.12,62.19)	0.05(-0.14,0.23)
United Arab Emirates	55.86(43.94,71.42)	0.39(0.29,0.54)	57.33(45.21,73.20)	3.67(2.69,4.99)	0.05(-0.39,0.48)
UK	59.42(46.73,76.11)	52.05(40.82,66.06)	52.60(41.23,67.52)	62.07(48.47,79.11)	-0.87(-1.47,-0.26)
Tanzania	18.28(14.11,23.64)	2.25(1.71,2.97)	17.27(13.37,22.31)	5.00(3.79,6.54)	-0.22(-0.26,-0.19)
USA	59.18(44.48,78.48)	182.95(137.44,241.57)	49.08(38.49,64.53)	249.95(193.92,324.01)	-1.06(-1.50,-0.62)
Virgin Islands US	37.38(28.81,49.06)	0.03(0.02,0.04)	33.26(25.52,43.33)	0.06(0.05,0.08)	-0.90(-1.63,-0.17)
Uruguay	56.01(43.83,71)	2.18(1.69,2.78)	48.24(37.65,61.79)	2.55(1.99,3.25)	-0.49(-0.63,-0.36)
Uzbekistan	85.12(68.80,106.73)	9.97(8.10,12.59)	80.01(64.42,100.03)	17.58(13.86,22.56)	-0.31(-0.44,-0.18)
Vanuatu	59.84(47.88,75.98)	0.04(0.03,0.05)	57.75(46.19,73.13)	0.10(0.08,0.12)	-0.12(-0.13,-0.11)
Venezuela	57.67(45.69,74.59)	5.91(4.70,7.65)	54.97(43.45,70.72)	16.18(12.78,20.96)	-0.26(-0.29,-0.23)
Vietnam	37.78(29.51,48.81)	15.21(11.87,19.82)	31.33(24.10,40.61)	28.48(21.87,37.26)	-0.48(-1.16,0.20)
Yemen	73.81(58.69,93.05)	3.95(3.12,5.05)	73.51(58.63,93.70)	11.07(8.75,14.29)	-0.14(-0.19,-0.08)
Zambia	18.34(14.22,23.64)	0.62(0.47,0.83)	17.15(13.38,22.18)	1.45(1.08,1.95)	-0.30(-0.42,-0.18)
Zimbabwe	54.73(42.69,69.33)	3.03(2.32,3.97)	55.62(43.34,71.01)	5.40(4.10,7.07)	0.15(0.07,0.23)

ASPR, age-standardized prevalence rate; No., number; UI, uncertainty interval; EAPC, estimated annual percentage change; CI, confidential interval.

Supplementary table 3. DALYs and age-standardized DALY rate per 1000 people for edentulism in 1990 and 2019, and its estimated annual percentage change in 204 countries and territories from 1990 to 2019.

	1990		2019		EAPC (95%CI)
	ASDR/1000 (95%UI)	DALYs*10^5 (95%UI)	ASDR/1000 (95%UI)	DALYs*10^5 (95%UI)	From 1990 to 2019
Afghanistan	2.02(1.29,2.99)	0.14(0.09,0.21)	1.98(1.28,2.93)	0.29(0.18,0.43)	-0.16(-0.26,-0.07)
Albania	1.99(1.27,2.92)	0.04(0.03,0.06)	1.76(1.12,2.59)	0.07(0.05,0.11)	-0.55(-0.60,-0.50)
Algeria	1.89(1.19,2.77)	0.25(0.16,0.36)	1.80(1.15,2.65)	0.64(0.41,0.95)	-0.25(-0.28,-0.21)
American Samoa	1.37(0.87,2.02)	0.00(0.00,0.00)	1.36(0.86,2.02)	0.00(0.00,0.00)	-0.04(-0.05,-0.03)
Andorra	1.38(0.87,2.07)	0.00(0.00,0.00)	1.30(0.83,1.94)	0.00(0.00,0.00)	-0.24(-0.28,-0.21)
Angola	1.03(0.66,1.53)	0.06(0.03,0.09)	0.93(0.59,1.38)	0.15(0.09,0.22)	-0.54(-0.62,-0.46)
Antigua and Barbuda	1.38(0.88,2.10)	0.00(0.00,0.00)	1.24(0.79,1.86)	0.00(0.00,0.00)	-0.39(-0.41,-0.37)
Argentina	1.45(0.91,2.16)	0.47(0.29,0.70)	1.25(0.79,1.86)	0.67(0.43,1.00)	-0.48(-0.52,-0.44)
Armenia	2.44(1.57,3.56)	0.07(0.04,0.10)	2.27(1.44,3.32)	0.09(0.06,0.14)	-0.45(-0.58,-0.31)
Australia	2.86(1.94,3.96)	0.55(0.37,0.76)	1.75(1.11,2.59)	0.68(0.43,1.00)	-0.67(-1.36,0.02)
Austria	1.84(1.18,2.71)	0.21(0.14,0.31)	1.64(1.05,2.41)	0.27(0.17,0.40)	-0.72(-1.14,-0.29)
Azerbaijan	2.33(1.50,3.40)	0.12(0.08,0.18)	2.16(1.38,3.14)	0.21(0.13,0.31)	-0.48(-0.66,-0.29)
Bahamas	1.29(0.82,1.94)	0.00(0.00,0.00)	1.21(0.77,1.83)	0.00(0.00,0.01)	-0.23(-0.26,-0.21)
Bahrain	1.67(1.07,2.43)	0.00(0.00,0.01)	1.58(1.01,2.34)	0.02(0.01,0.03)	-0.22(-0.25,-0.20)
Bangladesh	0.32(0.20,0.47)	0.14(0.09,0.21)	0.27(0.17,0.40)	0.34(0.21,0.50)	-0.56(-0.58,-0.54)
Barbados	1.39(0.88,2.09)	0.00(0.00,0.01)	1.29(0.81,1.93)	0.01(0.00,0.01)	-0.30(-0.32,-0.27)
Belarus	1.57(1.01,2.30)	0.20(0.13,0.29)	1.47(0.94,2.16)	0.22(0.14,0.33)	-0.32(-0.38,-0.26)
Belgium	1.88(1.20,2.73)	0.28(0.18,0.41)	1.66(1.06,2.45)	0.35(0.22,0.51)	-0.54(-0.80,-0.28)
Belize	1.49(0.94,2.22)	0.00(0.00,0.00)	1.34(0.85,2.03)	0.00(0.00,0.01)	-0.36(-0.39,-0.33)
Benin	0.81(0.52,1.20)	0.02(0.01,0.03)	0.76(0.49,1.14)	0.04(0.03,0.07)	-0.29(-0.33,-0.25)
Bermuda	1.29(0.82,1.94)	0.00(0.00,0.00)	1.17(0.74,1.75)	0.00(0.00,0.00)	-0.37(-0.40,-0.35)
Bhutan	0.50(0.32,0.74)	0.00(0.00,0.00)	0.42(0.26,0.63)	0.00(0.00,0.00)	-0.64(-0.65,-0.63)
Bolivia	2.90(1.86,4.20)	0.10(0.06,0.14)	2.65(1.71,3.85)	0.24(0.16,0.36)	-0.32(-0.34,-0.30)
Bosnia and Herzegovina	2.17(1.40,3.17)	0.09(0.06,0.13)	1.85(1.18,2.70)	0.11(0.07,0.16)	-1.07(-1.64,-0.50)
Botswana	1.72(1.10,2.59)	0.01(0.01,0.02)	1.53(0.97,2.33)	0.03(0.02,0.05)	-0.40(-0.41,-0.39)
Brazil	2.59(1.67,3.74)	2.35(1.50,3.44)	2.52(1.61,3.67)	6.01(3.83,8.78)	-0.08(-0.47,0.31)
Brunei	0.75(0.47,1.09)	0.00(0.00,0.00)	0.74(0.47,1.09)	0.00(0.00,0.00)	-0.07(-0.09,-0.04)
Bulgaria	1.70(1.08,2.50)	0.21(0.13,0.31)	1.51(0.96,2.24)	0.21(0.13,0.31)	-0.72(-0.98,-0.47)
Burkina Faso	0.54(0.34,0.82)	0.03(0.02,0.04)	0.50(0.31,0.76)	0.05(0.03,0.08)	-0.19(-0.47,0.10)
Burundi	0.52(0.33,0.78)	0.01(0.01,0.02)	0.53(0.33,0.80)	0.03(0.02,0.05)	0.06(0.03,0.09)
Cape Verde	0.80(0.50,1.19)	0.00(0.00,0.00)	0.70(0.44,1.04)	0.00(0.00,0.00)	-0.57(-0.61,-0.53)
Cambodia	1.34(0.85,1.97)	0.06(0.04,0.09)	1.15(0.74,1.71)	0.14(0.09,0.21)	-0.52(-0.58,-0.46)
Cameroon	0.75(0.48,1.12)	0.04(0.02,0.06)	0.73(0.46,1.08)	0.10(0.06,0.16)	-0.18(-0.24,-0.12)
Canada	1.21(0.76,1.81)	0.39(0.24,0.58)	0.98(0.62,1.45)	0.60(0.38,0.89)	-0.53(-0.81,-0.24)
Central African Republic	1.11(0.71,1.64)	0.02(0.01,0.03)	1.13(0.72,1.68)	0.03(0.02,0.05)	0.01(-0.01,0.03)
Chad	1.09(0.70,1.61)	0.03(0.02,0.05)	0.99(0.64,1.48)	0.07(0.04,0.10)	-0.39(-0.66,-0.13)
Chile	1.31(0.83,1.96)	0.13(0.08,0.19)	1.10(0.69,1.65)	0.26(0.16,0.40)	-0.58(-0.65,-0.51)
China	0.96(0.61,1.43)	7.57(4.82,11.47)	0.91(0.58,1.36)	17.56(11.28,26.49)	0.25(-0.43,0.93)
Colombia	1.32(0.84,1.95)	0.25(0.16,0.36)	1.19(0.76,1.78)	0.63(0.40,0.94)	-0.40(-0.43,-0.36)
Comoros	0.62(0.39,0.92)	0.00(0.00,0.00)	0.62(0.39,0.92)	0.00(0.00,0.00)	0.01(-0.11,0.14)
Congo	0.97(0.62,1.43)	0.01(0.01,0.02)	0.94(0.60,1.40)	0.03(0.02,0.05)	-0.19(-0.25,-0.12)
Cook Islands	1.43(0.92,2.13)	0.00(0.00,0.00)	1.31(0.84,1.94)	0.00(0.00,0.00)	-0.26(-0.28,-0.25)
Costa Rica	1.61(1.03,2.38)	0.03(0.02,0.04)	1.47(0.94,2.19)	0.08(0.05,0.11)	-0.33(-0.34,-0.32)

Croatia	1.78(1.14,2.60)	0.11(0.07,0.17)	1.57(1.00,2.33)	0.13(0.08,0.19)	-1.07(-1.65,-0.50)
Cuba	1.56(0.99,2.34)	0.16(0.10,0.24)	1.43(0.95,1.99)	0.27(0.18,0.37)	-0.38(-0.45,-0.31)
Cyprus	1.55(0.98,2.31)	0.01(0.01,0.02)	1.42(0.92,2.12)	0.03(0.02,0.04)	-0.31(-0.33,-0.28)
Czech	1.82(1.15,2.68)	0.25(0.15,0.36)	1.59(1.01,2.34)	0.31(0.20,0.47)	-0.61(-0.89,-0.33)
Cote d'Ivoire	1.05(0.68,1.55)	0.05(0.03,0.08)	1.03(0.66,1.53)	0.14(0.09,0.21)	-0.10(-0.24,0.04)
North Korea	0.81(0.52,1.22)	0.11(0.07,0.17)	0.91(0.58,1.37)	0.28(0.18,0.42)	0.40(0.37,0.44)
DR Congo	1.07(0.69,1.58)	0.22(0.14,0.33)	1.10(0.71,1.64)	0.56(0.35,0.84)	0.08(-0.03,0.19)
Denmark	1.42(0.90,2.13)	0.11(0.07,0.17)	1.21(0.77,1.82)	0.13(0.08,0.20)	-1.19(-2.11,-0.26)
Djibouti	0.46(0.29,0.69)	0.00(0.00,0.00)	0.46(0.29,0.70)	0.00(0.00,0.01)	0.00(-0.05,0.05)
Dominica	1.48(0.94,2.22)	0.00(0.00,0.00)	1.30(0.83,1.98)	0.00(0.00,0.00)	-0.5(-0.52,-0.47)
Dominican Republic	1.81(1.14,2.66)	0.07(0.04,0.10)	1.61(1.02,2.40)	0.15(0.09,0.22)	-0.42(-0.54,-0.29)
Ecuador	2.57(1.66,3.74)	0.14(0.09,0.21)	2.34(1.49,3.45)	0.36(0.23,0.53)	-0.51(-0.82,-0.19)
Egypt	1.97(1.25,2.91)	0.62(0.39,0.91)	1.82(1.15,2.67)	1.24(0.78,1.84)	-0.3(-0.32,-0.29)
El Salvador	1.69(1.07,2.50)	0.05(0.03,0.08)	1.56(0.98,2.30)	0.09(0.06,0.14)	-0.28(-0.28,-0.27)
Equatorial Guinea	1.08(0.69,1.60)	0.00(0.00,0.00)	0.77(0.49,1.15)	0.01(0.00,0.01)	-1.52(-1.72,-1.32)
Eritrea	0.52(0.33,0.79)	0.01(0.00,0.01)	0.49(0.31,0.75)	0.02(0.01,0.03)	-0.14(-0.20,-0.07)
Estonia	1.55(1.00,2.26)	0.03(0.02,0.05)	1.43(0.92,2.13)	0.03(0.02,0.05)	-0.75(-1.13,-0.37)
Eswatini	1.57(1.00,2.41)	0.01(0.00,0.01)	1.43(0.92,2.17)	0.01(0.01,0.02)	-0.33(-0.36,-0.31)
Ethiopia	0.53(0.33,0.80)	0.12(0.07,0.19)	0.50(0.31,0.76)	0.25(0.15,0.38)	-0.26(-0.49,-0.02)
Fiji	1.55(0.99,2.28)	0.01(0.00,0.01)	1.43(0.92,2.12)	0.01(0.01,0.02)	-0.24(-0.26,-0.23)
Finland	2.02(1.31,2.92)	0.14(0.09,0.21)	1.47(0.94,2.17)	0.16(0.11,0.25)	-1.60(-2.22,-0.97)
France	1.55(0.99,2.32)	1.25(0.80,1.86)	1.39(0.88,2.09)	1.73(1.10,2.62)	-0.33(-1.13,0.49)
Gabon	0.86(0.54,1.28)	0.01(0.00,0.01)	0.83(0.53,1.23)	0.01(0.01,0.02)	-0.11(-0.13,-0.09)
Gambia	0.81(0.51,1.20)	0.00(0.00,0.01)	0.78(0.50,1.17)	0.01(0.01,0.01)	-0.16(-0.20,-0.12)
Georgia	1.99(1.27,2.92)	0.12(0.08,0.18)	1.94(1.25,2.86)	0.11(0.07,0.16)	-0.30(-0.49,-0.11)
Germany	1.45(0.93,2.17)	1.79(1.14,2.67)	1.25(0.80,1.89)	2.20(1.42,3.29)	-1.00(-1.67,-0.33)
Ghana	0.73(0.46,1.08)	0.05(0.03,0.08)	0.66(0.42,0.99)	0.12(0.08,0.18)	-0.25(-0.41,-0.09)
Greece	1.59(1.01,2.36)	0.24(0.15,0.35)	1.46(0.94,2.16)	0.31(0.20,0.46)	-0.68(-1.21,-0.15)
Greenland	1.20(0.76,1.79)	0.00(0.00,0.00)	1.05(0.66,1.55)	0.00(0.00,0.00)	-0.50(-0.52,-0.48)
Grenada	1.48(0.93,2.21)	0.00(0.00,0.00)	1.29(0.82,1.93)	0.00(0.00,0.00)	-0.50(-0.51,-0.49)
Guam	1.29(0.82,1.94)	0.00(0.00,0.00)	1.26(0.80,1.88)	0.00(0.00,0.00)	-0.40(-0.69,-0.11)
Guatemala	1.93(1.22,2.80)	0.08(0.05,0.11)	1.82(1.17,2.65)	0.21(0.14,0.31)	-0.22(-0.23,-0.21)
Guinea	0.80(0.51,1.19)	0.03(0.02,0.04)	0.77(0.49,1.14)	0.05(0.03,0.07)	-0.16(-0.21,-0.12)
Guinea-Bissau	0.82(0.52,1.23)	0.00(0.00,0.01)	0.79(0.50,1.16)	0.01(0.00,0.01)	-0.16(-0.19,-0.12)
Guyana	1.51(0.97,2.26)	0.01(0.00,0.01)	1.35(0.87,2.02)	0.01(0.01,0.01)	-0.40(-0.41,-0.39)
Haiti	1.57(0.99,2.33)	0.05(0.03,0.08)	1.51(0.95,2.26)	0.11(0.07,0.16)	-0.15(-0.16,-0.15)
Honduras	1.73(1.10,2.53)	0.04(0.02,0.06)	1.62(1.02,2.39)	0.10(0.07,0.15)	-0.24(-0.25,-0.23)
Hungary	1.86(1.19,2.71)	0.27(0.17,0.39)	1.64(1.05,2.44)	0.30(0.19,0.45)	-0.32(-0.64,0.00)
Iceland	1.53(0.98,2.27)	0.00(0.00,0.01)	1.35(0.86,2.02)	0.01(0.00,0.01)	-0.51(-0.54,-0.48)
India	0.92(0.58,1.39)	4.05(2.53,6.22)	0.84(0.53,1.26)	9.40(5.89,14.27)	2.02(1.06,2.99)
Indonesia	1.36(0.87,2.01)	1.35(0.86,1.99)	1.23(0.78,1.82)	2.65(1.67,3.94)	-0.31(-0.36,-0.26)
Iran	1.52(0.96,2.24)	0.42(0.26,0.63)	1.44(0.90,2.12)	1.11(0.69,1.63)	-0.77(-1.34,-0.19)
Iraq	1.85(1.18,2.73)	0.15(0.10,0.23)	1.77(1.13,2.60)	0.45(0.29,0.67)	-0.30(-0.36,-0.24)
Ireland	2.14(1.38,3.11)	0.09(0.06,0.12)	1.75(1.12,2.57)	0.13(0.08,0.18)	-0.60(-0.95,-0.25)
Israel	1.82(1.17,2.67)	0.09(0.06,0.13)	1.61(1.03,2.37)	0.18(0.12,0.27)	-0.42(-0.46,-0.37)
Italy	1.39(0.89,2.06)	1.20(0.77,1.79)	1.33(0.85,1.98)	1.73(1.11,2.56)	-0.59(-1.08,-0.09)
Jamaica	1.48(0.94,2.21)	0.03(0.02,0.04)	1.37(0.86,2.05)	0.04(0.03,0.06)	-0.24(-0.26,-0.23)
Japan	1.25(0.81,1.84)	2.08(1.34,3.07)	1.04(0.66,1.54)	3.37(2.14,4.92)	-0.51(-1.32,0.30)
Jordan	1.94(1.24,2.86)	0.03(0.02,0.04)	1.84(1.16,2.70)	0.14(0.09,0.20)	-0.28(-0.32,-0.25)
Kazakhstan	2.56(1.64,3.74)	0.33(0.21,0.49)	2.38(1.52,3.47)	0.42(0.27,0.61)	-0.32(-0.45,-0.19)

Kenya	0.44(0.28,0.67)	0.04(0.03,0.06)	0.44(0.28,0.67)	0.12(0.07,0.18)	-0.39(-0.60,-0.17)
Kiribati	1.65(1.06,2.42)	0.00(0.00,0.00)	1.67(1.06,2.44)	0.00(0.00,0.00)	0.05(0.03,0.07)
Kuwait	1.67(1.06,2.45)	0.01(0.01,0.02)	1.59(1.01,2.34)	0.05(0.03,0.08)	-0.21(-0.24,-0.18)
Kyrgyzstan	2.37(1.51,3.45)	0.07(0.05,0.11)	2.36(1.50,3.46)	0.11(0.07,0.17)	-0.12(-0.25,0.02)
Laos	0.83(0.53,1.25)	0.02(0.01,0.03)	0.69(0.44,1.03)	0.03(0.02,0.05)	-0.68(-1.31,-0.06)
Latvia	1.23(0.78,1.81)	0.04(0.03,0.06)	1.17(0.75,1.75)	0.04(0.03,0.07)	-0.76(-1.29,-0.22)
Lebanon	1.92(1.23,2.84)	0.04(0.03,0.07)	1.79(1.15,2.62)	0.09(0.06,0.14)	-0.31(-0.33,-0.29)
Lesotho	2.00(1.27,3.06)	0.02(0.01,0.04)	1.80(1.16,2.72)	0.03(0.02,0.05)	-0.36(-0.37,-0.34)
Liberia	0.81(0.52,1.20)	0.01(0.01,0.01)	0.81(0.52,1.21)	0.02(0.01,0.03)	-0.23(-0.35,-0.11)
Libya	1.72(1.09,2.52)	0.03(0.02,0.05)	1.76(1.12,2.57)	0.10(0.06,0.15)	-0.02(-0.09,0.04)
Lithuania	1.26(0.81,1.86)	0.06(0.04,0.08)	1.15(0.74,1.74)	0.06(0.04,0.09)	-0.79(-1.19,-0.38)
Luxembourg	1.79(1.15,2.64)	0.01(0.01,0.01)	1.54(0.98,2.31)	0.01(0.01,0.02)	-0.88(-1.27,-0.49)
Madagascar	0.49(0.31,0.75)	0.03(0.02,0.04)	0.50(0.31,0.75)	0.07(0.04,0.10)	0.04(0.02,0.06)
Malawi	0.61(0.39,0.93)	0.03(0.02,0.04)	0.58(0.37,0.88)	0.05(0.03,0.08)	-0.18(-0.20,-0.17)
Malaysia	1.70(1.10,2.48)	0.17(0.11,0.24)	1.48(0.94,2.17)	0.4(0.25,0.60)	-0.83(-1.54,-0.11)
Maldives	1.11(0.71,1.65)	0.00(0.00,0.00)	0.97(0.62,1.44)	0.00(0.00,0.00)	-0.46(-0.50,-0.43)
Mali	0.64(0.41,0.96)	0.03(0.02,0.04)	0.59(0.37,0.88)	0.06(0.04,0.09)	-0.36(-0.65,-0.07)
Malta	1.35(0.86,2.03)	0.01(0.00,0.01)	1.14(0.73,1.73)	0.01(0.01,0.01)	-0.70(-0.82,-0.59)
Marshall Islands	1.60(1.02,2.33)	0.00(0.00,0.00)	1.52(0.98,2.24)	0.00(0.00,0.00)	-0.14(-0.15,-0.12)
Mauritania	1.05(0.67,1.56)	0.01(0.01,0.02)	0.99(0.64,1.48)	0.02(0.01,0.04)	-0.25(-0.46,-0.04)
Mauritius	1.78(1.15,2.61)	0.01(0.01,0.02)	1.55(1.00,2.27)	0.03(0.02,0.04)	-0.41(-1.00,0.18)
Mexico	1.85(1.18,2.70)	0.84(0.53,1.23)	1.78(1.13,2.60)	2.12(1.34,3.12)	-0.53(-0.79,-0.28)
Micronesia	1.62(1.04,2.37)	0.00(0.00,0.00)	1.55(1.00,2.28)	0.00(0.00,0.00)	-0.18(-0.19,-0.18)
Monaco	1.31(0.83,1.96)	0.00(0.00,0.00)	1.15(0.74,1.73)	0.00(0.00,0.00)	-0.44(-0.44,-0.43)
Mongolia	2.37(1.51,3.46)	0.03(0.02,0.04)	2.17(1.38,3.18)	0.05(0.03,0.08)	-0.41(-0.53,-0.30)
Montenegro	1.87(1.18,2.74)	0.01(0.01,0.02)	1.75(1.11,2.58)	0.02(0.01,0.02)	-0.34(-0.41,-0.27)
Morocco	2.26(1.45,3.32)	0.33(0.21,0.49)	2.06(1.32,3.00)	0.67(0.43,0.99)	-0.37(-0.42,-0.31)
Mozambique	0.56(0.36,0.84)	0.04(0.02,0.06)	0.51(0.33,0.77)	0.07(0.04,0.11)	-0.39(-0.41,-0.37)
Myanmar	0.83(0.53,1.25)	0.19(0.12,0.29)	0.66(0.41,1.00)	0.30(0.19,0.45)	-0.92(-1.80,-0.02)
Namibia	2.45(1.55,3.66)	0.02(0.01,0.03)	2.23(1.41,3.32)	0.04(0.03,0.06)	-0.33(-0.75,0.10)
Nauru	1.41(0.90,2.10)	0.00(0.00,0.00)	1.43(0.91,2.13)	0.00(0.00,0.00)	0.09(-0.05,0.23)
Nepal	0.39(0.24,0.58)	0.03(0.02,0.05)	0.32(0.20,0.48)	0.07(0.04,0.10)	-0.62(-0.84,-0.40)
Netherlands	2.25(1.45,3.27)	0.44(0.28,0.64)	1.97(1.27,2.89)	0.62(0.40,0.91)	-0.77(-1.35,-0.17)
New Zealand	2.69(1.71,4.02)	0.10(0.07,0.15)	1.97(1.24,2.93)	0.14(0.09,0.21)	-0.88(-1.25,-0.51)
Nicaragua	1.72(1.09,2.54)	0.03(0.02,0.04)	1.63(1.04,2.39)	0.08(0.05,0.11)	-0.23(-0.26,-0.21)
Niger	0.83(0.53,1.23)	0.03(0.02,0.04)	0.81(0.51,1.20)	0.07(0.05,0.11)	-0.12(-0.17,-0.07)
Nigeria	0.51(0.32,0.78)	0.24(0.15,0.36)	0.47(0.30,0.71)	0.46(0.28,0.70)	-0.64(-1.53,0.27)
Niue	1.49(0.95,2.21)	0.00(0.00,0.00)	1.39(0.89,2.07)	0.00(0.00,0.00)	-0.25(-0.27,-0.23)
North Macedonia	2.00(1.27,2.93)	0.04(0.02,0.06)	1.83(1.17,2.68)	0.06(0.04,0.09)	-0.39(-0.45,-0.33)
Northern Mariana Islands	1.30(0.84,1.93)	0.00(0.00,0.00)	1.31(0.84,1.94)	0.00(0.00,0.00)	0.06(0.03,0.09)
Norway	1.44(0.93,2.13)	0.09(0.06,0.14)	1.34(0.87,1.99)	0.12(0.08,0.18)	-0.20(-1.72,1.34)
Oman	1.68(1.07,2.47)	0.01(0.01,0.02)	1.58(1.01,2.32)	0.04(0.02,0.06)	-0.26(-0.28,-0.24)
Pakistan	0.80(0.51,1.18)	0.45(0.29,0.67)	0.77(0.49,1.14)	0.84(0.54,1.26)	-0.08(-0.87,0.71)
Palau	1.45(0.93,2.14)	0.00(0.00,0.00)	1.37(0.88,2.03)	0.00(0.00,0.00)	-0.18(-0.20,-0.16)
Palestine	2.06(1.32,3.04)	0.02(0.01,0.03)	1.89(1.20,2.80)	0.05(0.03,0.08)	-0.31(-0.33,-0.29)
Panama	1.60(1.01,2.36)	0.02(0.02,0.04)	1.42(0.90,2.10)	0.06(0.04,0.09)	-0.43(-0.45,-0.40)
Papua New Guinea	1.62(1.03,2.39)	0.03(0.02,0.04)	1.55(0.99,2.29)	0.07(0.05,0.11)	-0.12(-0.15,-0.10)
Paraguay	1.97(1.26,2.92)	0.04(0.03,0.06)	1.76(1.11,2.60)	0.10(0.06,0.14)	-0.12(-0.73,0.49)
Peru	3.00(1.94,4.35)	0.38(0.24,0.54)	2.76(1.76,4.02)	0.89(0.57,1.29)	-0.18(-0.27,-0.10)

Philippines	1.79(1.15,2.63)	0.56(0.36,0.82)	1.72(1.10,2.53)	1.36(0.87,2.01)	-0.13(-0.16,-0.09)
Poland	2.21(1.41,3.23)	0.96(0.61,1.41)	1.52(0.97,2.23)	1.00(0.63,1.47)	-1.27(-1.87,-0.67)
Portugal	1.59(1.03,2.35)	0.22(0.14,0.32)	1.41(0.90,2.12)	0.31(0.20,0.46)	-0.47(-0.58,-0.36)
Puerto Rico	1.29(0.82,1.94)	0.05(0.03,0.07)	1.17(0.75,1.79)	0.08(0.05,0.12)	-0.65(-1.01,-0.28)
Qatar	1.53(0.97,2.25)	0.00(0.00,0.00)	1.45(0.92,2.14)	0.02(0.01,0.04)	-0.29(-0.33,-0.25)
South Korea	0.84(0.54,1.24)	0.25(0.16,0.37)	0.71(0.45,1.06)	0.61(0.39,0.91)	-0.86(-2.01,0.30)
Moldova	1.50(0.95,2.22)	0.07(0.04,0.10)	1.53(0.98,2.26)	0.09(0.05,0.13)	0.02(-0.09,0.12)
Romania	1.66(1.06,2.46)	0.46(0.29,0.69)	1.45(0.92,2.16)	0.51(0.32,0.77)	-0.82(-1.13,-0.51)
Russia	1.94(1.25,2.84)	3.42(2.19,5.01)	1.91(1.23,2.80)	4.32(2.76,6.35)	-0.09(-0.15,-0.03)
Rwanda	0.53(0.33,0.78)	0.02(0.01,0.03)	0.50(0.32,0.75)	0.04(0.02,0.06)	-0.22(-0.28,-0.16)
Saint Kitts and Nevis	1.39(0.89,2.09)	0.00(0.00,0.00)	1.22(0.77,1.84)	0.00(0.00,0.00)	-0.46(-0.48,-0.43)
Saint Lucia	1.44(0.91,2.15)	0.00(0.00,0.00)	1.31(0.82,1.96)	0.00(0.00,0.00)	-0.35(-0.36,-0.33)
Saint Vincent and the Grenadines	1.48(0.94,2.21)	0.00(0.00,0.00)	1.29(0.82,1.95)	0.00(0.00,0.00)	-0.51(-0.54,-0.49)
Samoa	1.62(1.04,2.38)	0.00(0.00,0.00)	1.55(1.01,2.27)	0.00(0.00,0.00)	-0.19(-0.21,-0.18)
San Marino	1.47(0.94,2.20)	0.00(0.00,0.00)	1.34(0.86,2.01)	0.00(0.00,0.00)	-0.38(-0.44,-0.32)
Sao Tome and Principe	0.77(0.49,1.15)	0.00(0.00,0.00)	0.72(0.46,1.08)	0.00(0.00,0.00)	-0.29(-0.35,-0.24)
Saudi Arabia	1.62(1.04,2.38)	0.11(0.07,0.17)	1.55(0.99,2.28)	0.36(0.22,0.55)	-0.18(-0.20,-0.16)
Senegal	1.12(0.71,1.66)	0.04(0.03,0.06)	1.06(0.68,1.56)	0.09(0.06,0.14)	-0.23(-0.42,-0.04)
Serbia	1.88(1.19,2.77)	0.21(0.13,0.31)	1.74(1.11,2.58)	0.26(0.17,0.40)	-0.37(-0.43,-0.31)
Seychelles	1.05(0.67,1.56)	0.00(0.00,0.00)	0.93(0.59,1.39)	0.00(0.00,0.00)	-0.37(-0.41,-0.34)
Sierra Leone	0.83(0.53,1.23)	0.02(0.01,0.03)	0.80(0.50,1.18)	0.03(0.02,0.05)	-0.17(-0.25,-0.09)
Singapore	0.79(0.51,1.18)	0.02(0.01,0.03)	0.72(0.45,1.07)	0.06(0.03,0.08)	-0.32(-0.33,-0.31)
Slovakia	1.78(1.13,2.61)	0.11(0.07,0.16)	1.52(0.96,2.26)	0.14(0.09,0.20)	-0.55(-0.87,-0.23)
Slovenia	2.12(1.37,3.09)	0.05(0.03,0.08)	1.88(1.21,2.77)	0.08(0.05,0.11)	-0.89(-1.31,-0.46)
Solomon Islands	1.70(1.08,2.48)	0.00(0.00,0.00)	1.63(1.04,2.42)	0.00(0.00,0.01)	-0.10(-0.13,-0.08)
Somalia	0.59(0.37,0.89)	0.02(0.01,0.03)	0.62(0.39,0.92)	0.05(0.03,0.08)	0.16(0.12,0.19)
South Africa	2.16(1.37,3.21)	0.57(0.36,0.85)	1.64(1.05,2.45)	0.84(0.53,1.26)	-0.99(-1.43,-0.55)
South Sudan	0.40(0.25,0.60)	0.01(0.01,0.02)	0.41(0.26,0.61)	0.02(0.01,0.03)	0.05(0.04,0.06)
Spain	1.48(0.94,2.11)	0.81(0.52,1.16)	1.00(0.64,1.52)	0.89(0.57,1.33)	-2.00(-3.15,-0.84)
Sri Lanka	1.03(0.66,1.52)	0.11(0.07,0.17)	0.88(0.56,1.31)	0.22(0.14,0.33)	-1.50(-2.53,-0.46)
Sudan	2.05(1.30,3.04)	0.20(0.13,0.30)	1.89(1.21,2.78)	0.40(0.25,0.58)	-0.34(-0.36,-0.31)
Suriname	1.42(0.90,2.13)	0.00(0.00,0.01)	1.32(0.84,1.98)	0.01(0.01,0.01)	-0.32(-0.34,-0.29)
Sweden	1.21(0.78,1.80)	0.17(0.11,0.25)	1.25(0.80,1.88)	0.24(0.15,0.36)	-0.45(-1.18,0.29)
Switzerland	1.26(0.85,1.75)	0.13(0.09,0.18)	1.04(0.66,1.56)	0.17(0.11,0.25)	-1.16(-1.65,-0.67)
Syria	2.03(1.30,2.99)	0.12(0.07,0.17)	1.92(1.24,2.84)	0.25(0.16,0.37)	-0.28(-0.33,-0.24)
Taiwan (Province of China)	0.67(0.43,0.99)	0.10(0.06,0.15)	0.68(0.43,1.02)	0.27(0.17,0.40)	0.12(0.08,0.15)
Tajikistan	2.43(1.55,3.54)	0.07(0.05,0.10)	2.36(1.52,3.47)	0.13(0.08,0.19)	-0.23(-0.39,-0.07)
Thailand	1.15(0.73,1.69)	0.42(0.26,0.62)	0.99(0.63,1.48)	0.99(0.63,1.50)	-0.51(-0.53,-0.49)
Timor-Leste	1.20(0.76,1.78)	0.00(0.00,0.01)	1.06(0.67,1.58)	0.01(0.01,0.01)	-0.49(-0.54,-0.44)
Togo	0.81(0.51,1.21)	0.01(0.01,0.02)	0.79(0.50,1.18)	0.03(0.02,0.05)	-0.10(-0.14,-0.06)
Tokelau	1.60(1.03,2.37)	0.00(0.00,0.00)	1.47(0.94,2.17)	0.00(0.00,0.00)	-0.33(-0.34,-0.31)
Tonga	1.62(1.04,2.37)	0.00(0.00,0.00)	1.51(0.97,2.25)	0.00(0.00,0.00)	-0.21(-0.22,-0.20)
Trinidad and Tobago	1.39(0.89,2.08)	0.01(0.01,0.02)	1.22(0.78,1.85)	0.02(0.01,0.03)	-0.59(-0.64,-0.54)
Tunisia	1.94(1.24,2.85)	0.10(0.07,0.15)	1.76(1.12,2.59)	0.23(0.14,0.33)	-0.40(-0.59,-0.22)
Turkey	2.45(1.56,3.58)	0.94(0.60,1.38)	2.18(1.40,3.21)	1.97(1.26,2.89)	-0.33(-0.54,-0.12)
Turkmenistan	2.29(1.46,3.36)	0.05(0.03,0.07)	2.08(1.32,3.05)	0.08(0.05,0.12)	-0.48(-0.64,-0.31)
Tuvalu	1.66(1.07,2.44)	0.00(0.00,0.00)	1.54(0.99,2.27)	0.00(0.00,0.00)	-0.22(-0.24,-0.21)
Uganda	0.52(0.33,0.78)	0.04(0.02,0.06)	0.49(0.31,0.73)	0.09(0.05,0.13)	-0.25(-0.26,-0.24)

Ukraine	1.85(1.18,2.71)	1.28(0.82,1.90)	1.85(1.19,2.71)	1.35(0.86,1.98)	0.08(-0.12,0.27)
United Arab Emirates	1.53(0.97,2.27)	0.01(0.01,0.02)	1.57(1.00,2.32)	0.10(0.06,0.16)	0.03(-0.40,0.46)
UK	1.63(1.04,2.40)	1.42(0.90,2.10)	1.44(0.92,2.13)	1.68(1.07,2.50)	-0.88(-1.49,-0.26)
Tanzania	0.50(0.32,0.75)	0.06(0.04,0.09)	0.48(0.30,0.72)	0.14(0.09,0.21)	-0.20(-0.23,-0.16)
USA	1.60(0.98,2.41)	4.92(3.04,7.42)	1.32(0.84,1.97)	6.69(4.24,9.85)	-1.07(-1.51,-0.62)
Virgin Islands US	1.03(0.64,1.56)	0.00(0.00,0.00)	0.91(0.57,1.38)	0.00(0.00,0.00)	-0.93(-1.65,-0.20)
Uruguay	1.54(0.96,2.28)	0.06(0.04,0.09)	1.32(0.84,1.97)	0.07(0.04,0.10)	-0.50(-0.64,-0.37)
Uzbekistan	2.36(1.52,3.45)	0.28(0.18,0.41)	2.21(1.41,3.26)	0.49(0.31,0.73)	-0.32(-0.45,-0.19)
Vanuatu	1.63(1.04,2.41)	0.00(0.00,0.00)	1.57(1.00,2.30)	0.00(0.00,0.00)	-0.13(-0.15,-0.12)
Venezuela	1.58(1.00,2.35)	0.16(0.10,0.24)	1.51(0.97,2.22)	0.45(0.29,0.66)	-0.25(-0.28,-0.22)
Vietnam	1.04(0.67,1.54)	0.42(0.27,0.63)	0.86(0.55,1.29)	0.79(0.50,1.19)	-0.48(-1.15,0.20)
Yemen	2.03(1.29,2.97)	0.11(0.07,0.16)	2.02(1.28,2.97)	0.31(0.19,0.46)	-0.13(-0.18,-0.08)
Zambia	0.50(0.32,0.77)	0.02(0.01,0.03)	0.47(0.30,0.72)	0.04(0.02,0.06)	-0.29(-0.41,-0.18)
Zimbabwe	1.53(0.98,2.32)	0.09(0.05,0.13)	1.54(0.98,2.35)	0.15(0.09,0.24)	0.14(0.06,0.22)

ASDR, age-standardized disability-adjusted life years rate; DALYs, disability-adjusted life years; No., number; UI, uncertainty interval; EAPC, estimated annual percentage change; CI, confidential interval.

1.2 Supplementary Figures

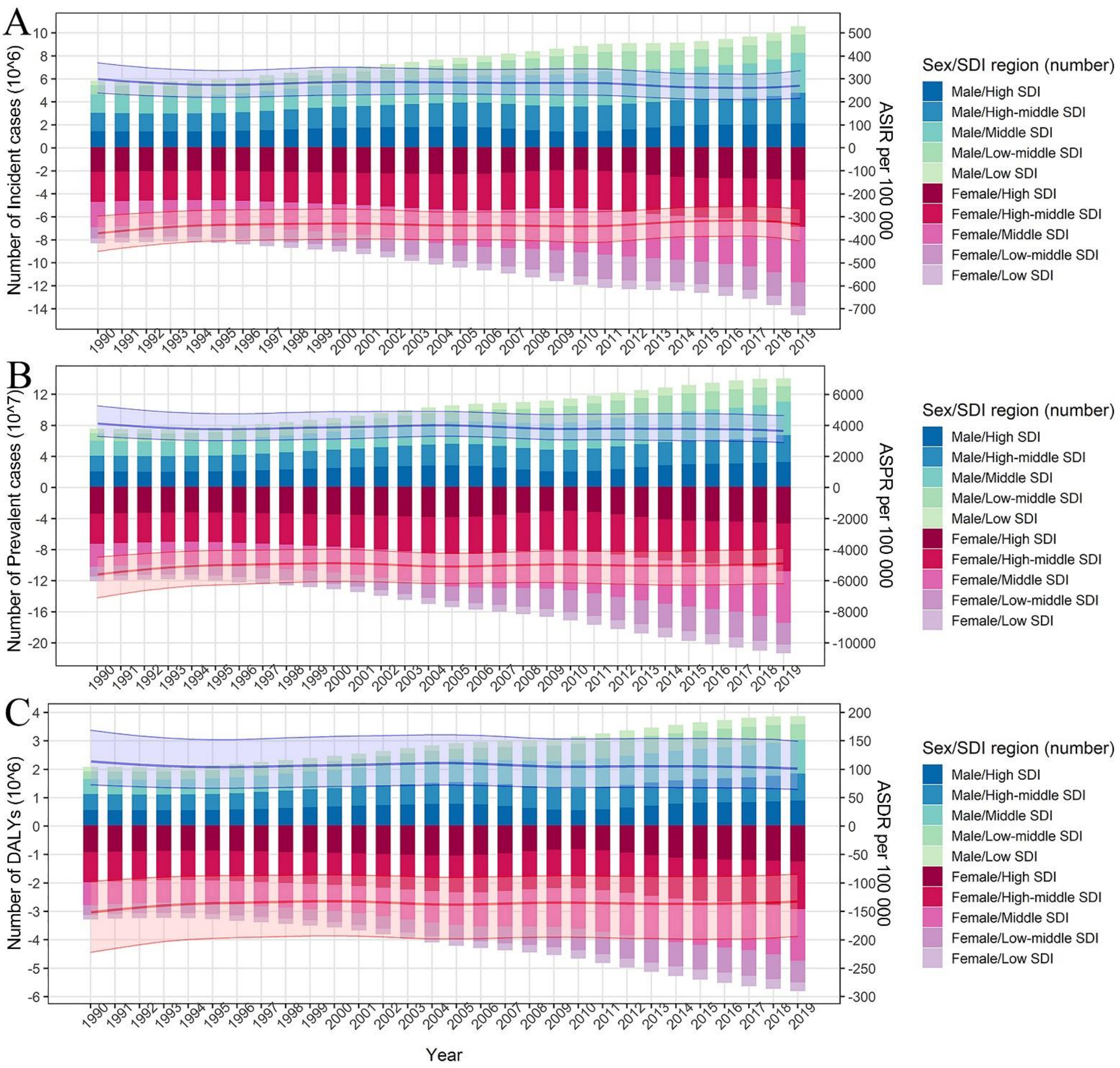

Supplementary figure 1. Incident cases, prevalent cases, DALYs and the corresponding ASRs of edentulism by sex, year, and SDI regions. (A) incident cases and ASIR (B) prevalent cases and ASPR; (B) DALYs and ASDR. ASIR: age-standardized incidence rate; ASPR: age-standardized prevalence rate; DALYs: disability-adjusted life years; ASDR: age-standardized DALY rate; SDI: socio-demographic index; ASRs: age-standardized rates.

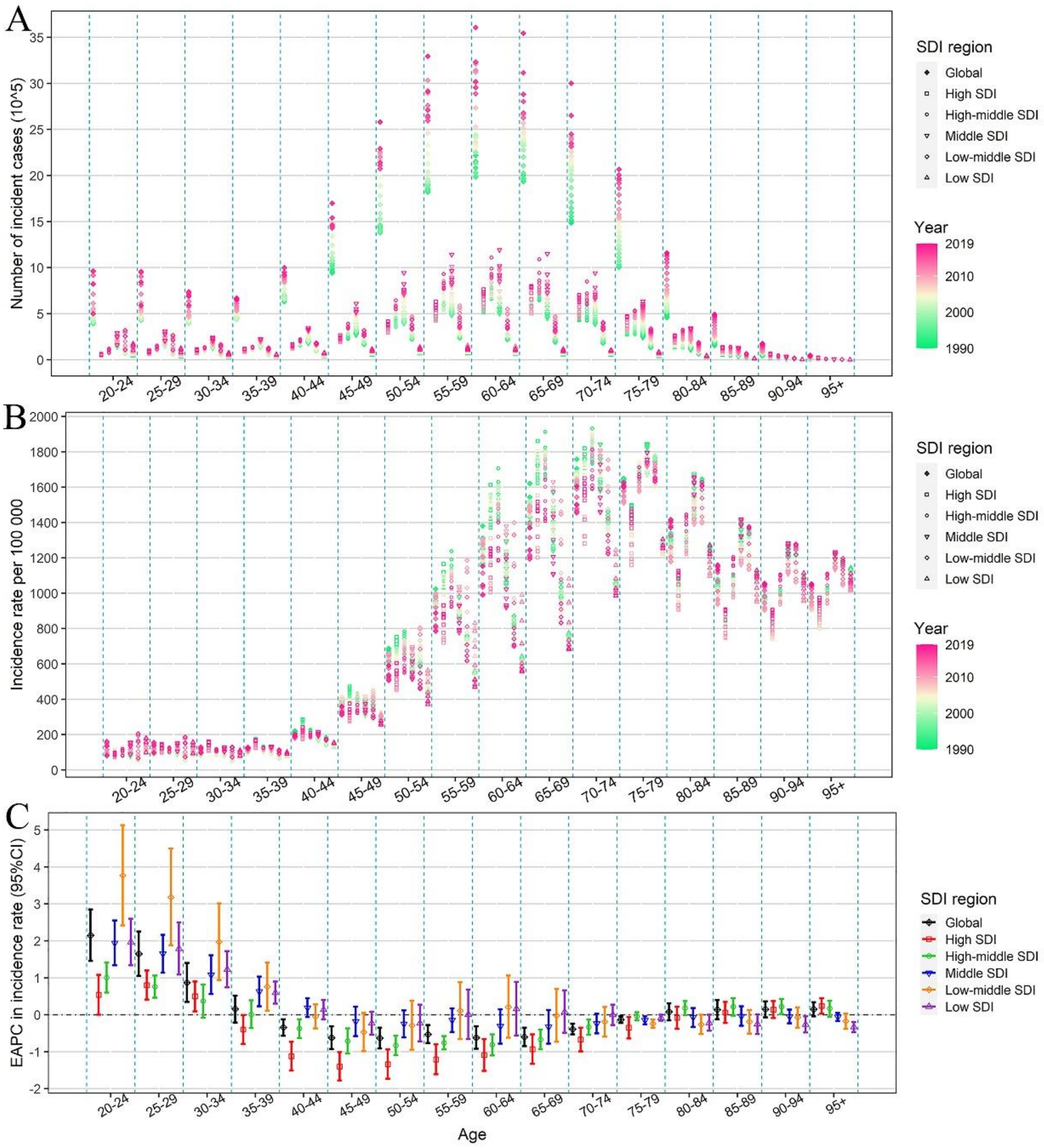

Supplementary figure 2. Incident cases, incidence rate, and EAPC of incidence rate of edentulism by age group, and SDI regions from 1990 to 2019. (A) incident cases; (B) incidence rate; (C)EAPC of incidence rate. SDI: socio-demographic index; EAPC: estimated annual percentage change.

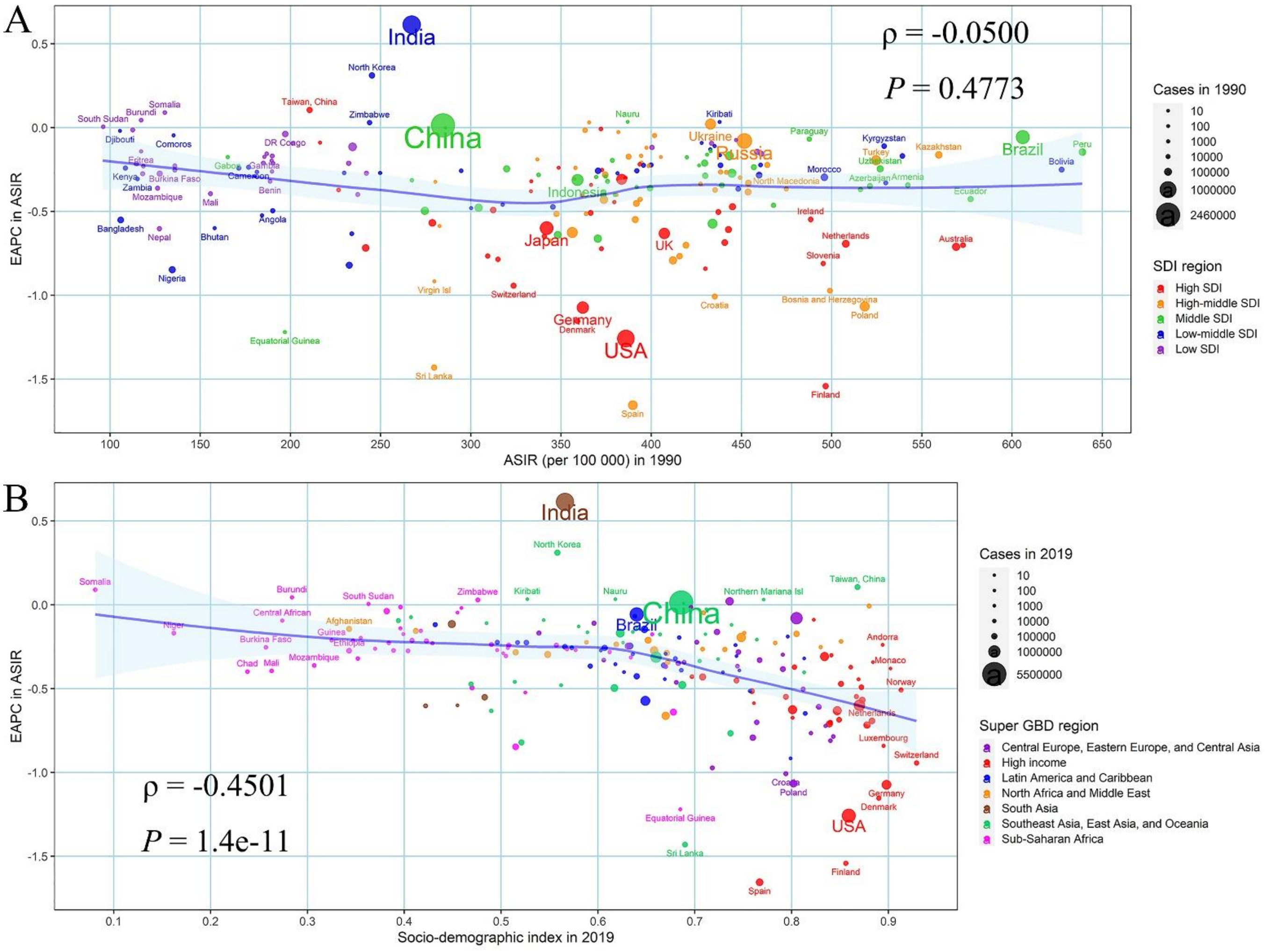

Supplementary figure 3. The association between ASIR in 1990, SDI in 2019, and the EAPC of ASIR from 1990 to 2019. (A) ASIR in 1990 and the EAPC of ASIR from 1990 to 2019; (B) SDI in 2019 and the EAPC of ASIR from 1990 to 2019. The blue line was an adaptive association fitted with adaptive Loess regression based on all data points. EAPC: estimated annual percentage change; SDI: socio-demographic index; ASIR: age-standardized incidence rate.

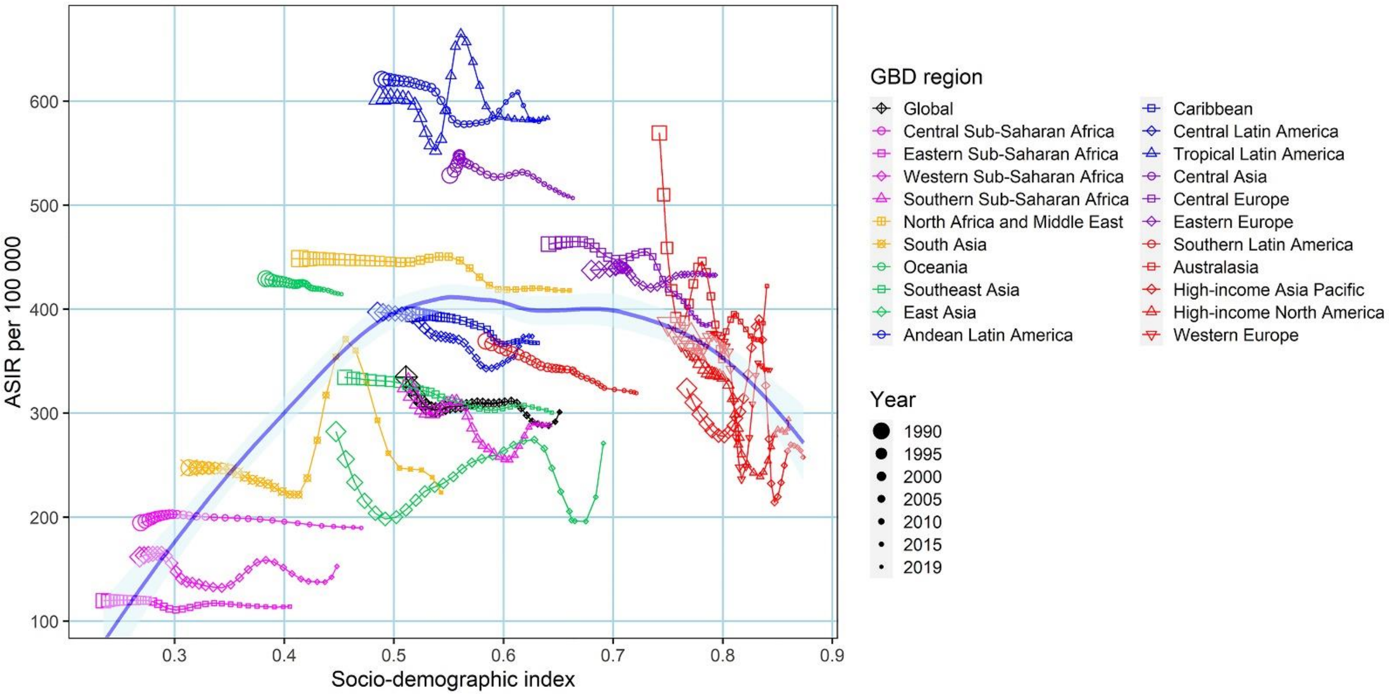

Supplementary figure 4. The association between ASIR, and SDI by GBD regions from 1990 to 2019. The blue line was an adaptive association fitted with adaptive Loess regression based on all data points. GBD: Global Burden of Disease; ASIR: age-standardized incidence rate; SDI: socio-demographic index.
